# Site‐Selective Coordination Assembly of Dynamic Metal‐Phenolic Networks

**DOI:** 10.1002/anie.202208037

**Published:** 2022-07-13

**Authors:** Wanjun Xu, Shuaijun Pan, Benjamin B. Noble, Jingqu Chen, Zhixing Lin, Yiyuan Han, Jiajing Zhou, Joseph J. Richardson, Irene Yarovsky, Frank Caruso

**Affiliations:** ^1^ Department of Chemical Engineering The University of Melbourne Parkville Victoria 3010 Australia; ^2^ State Key Laboratory of Chemo/Biosensing and Chemometrics College of Chemistry and Chemical Engineering Hunan University Changsha 410082 China; ^3^ School of Engineering RMIT University Melbourne Victoria 3001 Australia; ^4^ Department of Materials Engineering The University of Tokyo Tokyo 113-8656 Japan

**Keywords:** Flavonoids, Metal-Organic Frameworks, Microcapsules, Site-Selective Assembly, Supramolecular Chemistry

## Abstract

Coordination states of metal‐organic materials are known to dictate their physicochemical properties and applications in various fields. However, understanding and controlling coordination sites in metal‐organic systems is challenging. Herein, we report the synthesis of site‐selective coordinated metal‐phenolic networks (MPNs) using flavonoids as coordination modulators. The site‐selective coordination was systematically investigated experimentally and computationally using ligands with one, two, and multiple different coordination sites. Tuning the multimodal Fe coordination with catechol, carbonyl, and hydroxyl groups within the MPNs enabled the facile engineering of diverse physicochemical properties including size, selective permeability (20–2000 kDa), and pH‐dependent degradability. This study expands our understanding of metal‐phenolic chemistry and provides new routes for the rational design of structurally tailorable coordination‐based materials.

## Graphical abstract

The site‐selective coordination assembly of metal‐phenolic capsules results in distinct cross‐linking density and structures, which enables control over the physicochemical properties of the capsules, including thickness, permeability, size, and pH‐dependent degradability. Our work provides a pathway for the design of structurally tunable coordination materials.

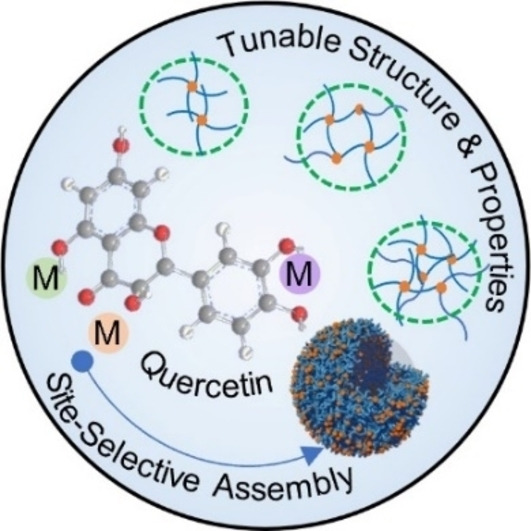

## Introduction

Coordination‐driven self‐assembly is a versatile supramolecular strategy for the construction of functional molecular architectures.^[1]^ In particular, supramolecular metal‐organic network materials have attracted widespread scientific and industrial interest owing to their porous structure and hybrid physicochemical properties arising from the metal and organic ligand components.^[2]^ Metal‐phenolic networks (MPNs), stabilized by the chelation between metal ions (e.g., Fe^II^, Zn^II^, Fe^III^) and phenolic ligands (e.g., tannic acid (TA), epigallocatechin gallate), are promising coordination materials for bionanotechnology and nanoarchitectonics.^[3]^ The vast choice of available building blocks enables the engineering of MPNs with a high degree of modularity and versatility, and their subsequent broad application in catalysis, gas storage, molecule separation, and bioimaging.^[4]^ In particular, the dynamic and multivalent transition of MPNs between different coordination states (e.g., mono‐, bis‐, and tris‐state) provides a viable pathway for exploiting supramolecular interactions and interfacial properties.^[5]^ In this context, the formation of existing MPNs primarily relies on catechol or galloyl groups for coordination, where acidic phenolic ligands such as TA are mostly used,^[6]^ whereas the integration of other coordination motifs is rarely explored.

Flavonoids, a major class of polyphenols, have attracted attention owing to their extensive biological activities such as antioxidant, antiviral, antibacterial, and anticancer activities.^[7]^ The abundant catechol groups, as well as the additional carbonyl and hydroxyl groups, provide multiple moieties for metal coordination that may lead to distinct formation mechanisms and structural dynamics in MPN materials. For example, flavonoids were previously used for engineering biofunctional metal‐coordinated films^[8]^ but the diverse binding chemistry was not explored in detail. A deeper understanding of the use of flavonoids in MPN engineering would improve MPN design and function and shed light into modulating the chelation state in metal‐organic materials. Specifically, a comprehensive understanding of coordination anisotropy in phenolic ligands is expected to expand the possibilities of MPNs to achieve advanced functionalities for broader applications.^[9]^

**Figure 1:**
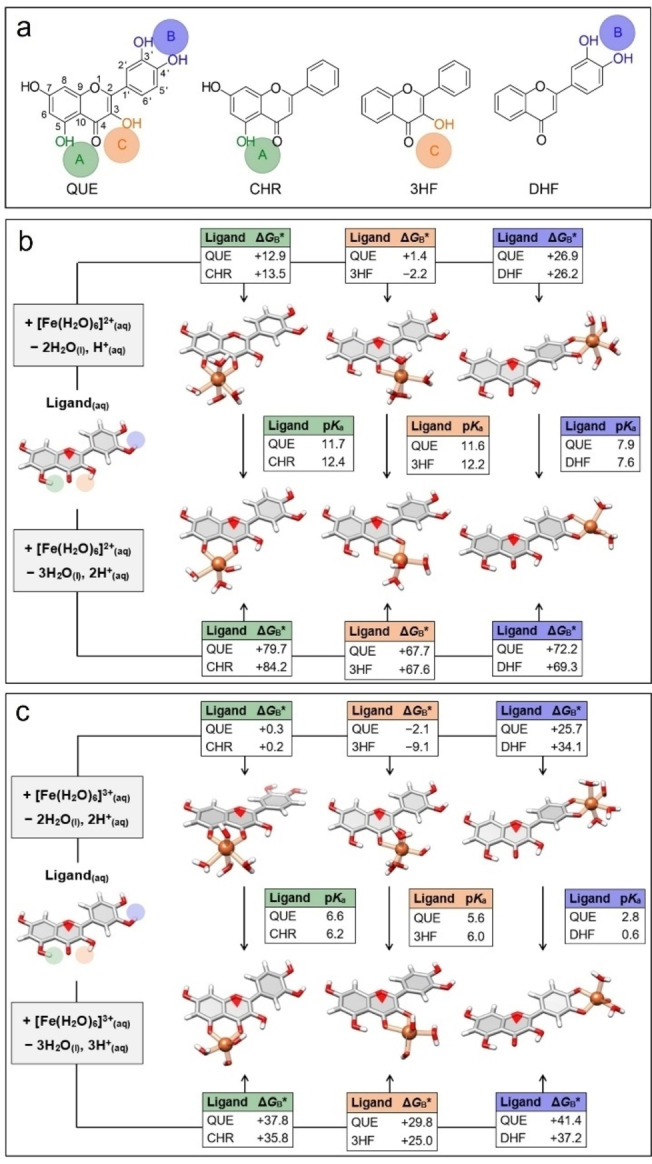
a) Chemical structures of flavonoids investigated: quercetin (QUE), chrysin (CHR), 3‐hydroxyflavone (3HF), and 3′,4′‐dihydroxyflavone (DHF). The green (Site A), violet (Site B), and orange (Site C) circles represent acetylacetone‐, catechol‐, and maltol‐based coordination sites. b), c) Optimized solution‐phase structures of the various Fe^II^‐QUE complexes (b) and Fe^III^‐QUE complexes (c) and their corresponding Gibbs free energies for binding and deprotonation (in kJ mol^−1^, calculated at 25 °C and pH 0). Respective p*K*_a_ values for the resulting complexes and Gibbs free energies for the control flavonoids are also given. Calculations were performed at the DLPNO‐CCSD(T)/cc‐pVTZ//B97M‐V/def2‐TZVP level of theory with conductor‐like polarizable continuum model (CPCM) solvation corrections.

Herein, we investigate the structural dynamics and selective coordination modes of MPNs both computationally and experimentally using a series of carbonyl‐containing flavonoids as phenolic ligands in a comparison study (Figure 1, Figure S1). Quercetin (QUE) was used as a model ligand, as its three potential binding sites (i.e., acetylacetone, maltol, and catechol) can selectively coordinate with metal ions (e.g., Fe^II^, Fe^III^), allowing for a systematic and mechanistic study of network formation and stability. Density functional theory and wavefunction calculations showed that maltol groups displayed the highest binding affinity for Fe^II^ and Fe^III^, followed by acetylacetone groups, and finally catechol groups. In addition, spectroscopic measurements indicated that the dominant coordination mode of assembly at pH<7 was metal‐maltol coordination, whereas metal‐catechol coordination became significant at pH>7. Furthermore, an increase in assembly pH from 1 to 12 resulted in a switch of the coordination state of the catechol groups from the mono‐state to the bis‐/tris‐state. Finally, the pH‐dependent site selection between maltol and catechol groups, along with the multivalent state transition displayed by Fe‐catechol coordination, gave rise to tailorable MPN microstructures. Specifically, MPN capsules were obtained with tunable physicochemical properties, including dynamic size, pH‐responsive permeability, and coordination‐based disassembly kinetics. The current site‐selective coordination strategy expands the realm of MPN assembly and offers opportunities for constructing advanced metal‐organic materials with tunable properties.

## Results and Discussion

**Scheme 1:**
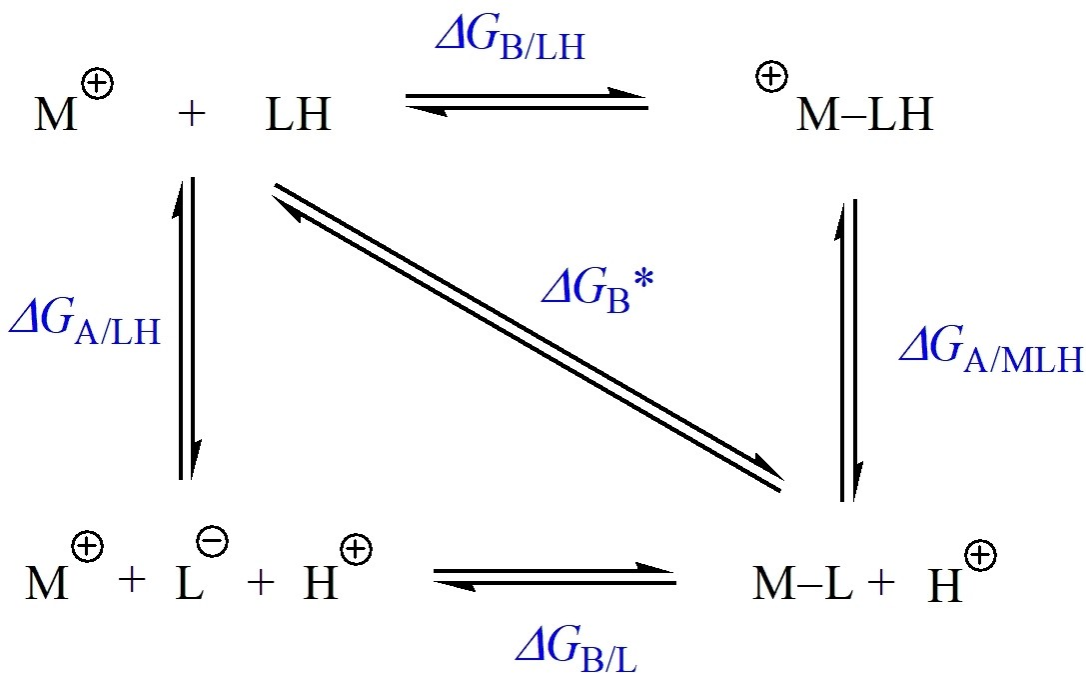
Hess's Law thermocycle for the coordination of a ligand (LH) to a metallic species (M^+^). Δ*G*_B/LH_ and Δ*G*_B/L_ define the strength of binding of the (protonated) ligand and its conjugate base, respectively. Δ*G*_A/LH_ and Δ*G*_A/MLH_ define the energy of the free ligand and complexed ligand, respectively. Δ*G*_B_* encompasses both binding and deprotonation processes.

Site‐selective coordination between Fe^II^ or Fe^III^ and different ligands was first studied using high‐level quantum chemistry, and their Gibbs free binding energies (Δ*G*) and p*K*_a_ values were predicted from ab initio calculations. The fundamental thermodynamics that govern the coordination of organic ligands to metallic species are defined in Scheme 1. Given the respective p*K*_a_ values of the flavonoid ligands and conditions of interest (e.g., pH 4–9) for coordination assembly, Δ*G*_B_ *, which is the Gibbs free energy that describes the strength of ligand binding as a function of [H^+^] (i.e., pH), was used to assess metal‐ligand coordination within the MPNs.

To assess the interference of different binding coordinate sites on selective binding, QUE, which contains three different coordination sites, was chosen as a model ligand and three types of flavonoids that contain one of the binding sites present in QUE were chosen as control ligands (Figure 1a). The three flavonoids are 3‐hydroxyflavone (3HF; with only a maltol group), chrysin (CHR; with only an acetylacetone group), and 3′,4′‐dihydroxyflavone (DHF; with only a catechol group). The fundamental binding energetics for these ligands to Fe^II^ and Fe^III^ were then calculated using high‐level quantum chemistry using ORCA 5.0.1.^[10]^ The calculations predicted that the control flavonoids (3HF, CHR, and DHF) had very similar binding energetics to the respective maltol (orange), acetylacetone (green), and catechol (violet) sites of QUE (Figure 1b and c, Tables S1–S8). Site selectivity was predicted for Fe^II^‐QUE complexes, with binding at the maltol group being favored by around 12 kJ mol^−1^ over binding at the acetylacetone group regardless of pH (Figure 1b). This energy difference corresponds to a selectivity of approximately 100 : 1 between these two sites. Binding of Fe^II^ to the catechol moiety of QUE was comparatively weak compared with binding to the maltol group, being around 25 kJ mol^−1^ less favorable at low‐neutral pH (i.e., <7). Strong complexation of the catechol moiety of QUE to Fe^II^ would only be anticipated at pH 7–9, where the effective Δ*G*_B_* was around −15 to −35 kJ mol^−1^ (Table S4). In contrast to acetylacetone binding, catechol complexation would not directly interfere with binding to the maltol group.

As depicted in Figure 1c, flavonoid binding to Fe^III^ was considerably more thermodynamically favorable than binding to Fe^II^, with significant contractions of the Fe−O bonds observed upon oxidation. At low pH (≈4), the calculations predicted that Fe^III^‐QUE complexes displayed poor selectivity for maltol than for acetylacetone sites. However, deprotonation of the water coligands around Fe^III^‐QUE complexes enhanced maltol site selectivity (Figure 1c, Figures S2 and S3). A predicted selectivity of around 7 kJ mol^−1^ was obtained at pH 7, which corresponds to approximately 95 % selectivity for maltol (versus acetylacetone) binding (Table S8). Ionization of these coligands was accompanied by a corresponding elongation of the respective Fe−O chelate bonds, suggesting that steric factors are (at least partially) responsible for controlling maltol vs acetylacetone site selectivity. As with Fe^II^, catechol coordination for Fe^III^‐Que was predicted to be less favorable than either maltol or acetylacetone binding. However, this binding was still highly favorable in the pH range of 4–9, with an effective Δ*G*_B_* between −25 to −120 kJ mol^−1^. By comparing the predicted QUE site selectivity for Fe^II^ and Fe^III^ as a function of pH (Figures S4 and S5, Table S9), we found that Fe^II^ displayed an effective binding preference for maltol over acetylacetone sites of QUE in the entire pH range (i.e., 0–14). To simply clarify the site selectivity among conditions of interest (e.g., pH 4–9), we chose Fe^II^ as the main metal ion for the following study. The calculations indicated that simultaneous coordination through the maltol and catechol moieties in QUE would underpin the structure of Fe^II^‐QUE networks.

Subsequently, these differences in binding site coordination to Fe^II^ were observed experimentally. For example, a bathochromic shift in the peak from 350 nm (for free DHF) to 390 nm (for Fe^II^‐DHF complexes) at pH 7 and 9 indicated Fe‐catechol coordination (Figure S6a). The presence of the band at 480 nm for Fe^II^‐3HF complexes at pH 4, 7, and 9 suggested Fe‐maltol coordination (Figure S6b). Finally, coordination between the acetylacetone groups (i.e., CHR) and Fe^II^ occurred at pH 9, as indicated by a bathochromic shift (Figure S6c). In the model ligand QUE, coordination is unlikely to occur at the competing acetylacetone group owing to steric hindrance from the coordinated maltol group.^[11]^ Collectively, these results demonstrate that Fe‐flavonoid coordination is primarily mediated by maltol and catechol groups, where the dominant coordination site can be controlled by pH.

**Figure 2:**
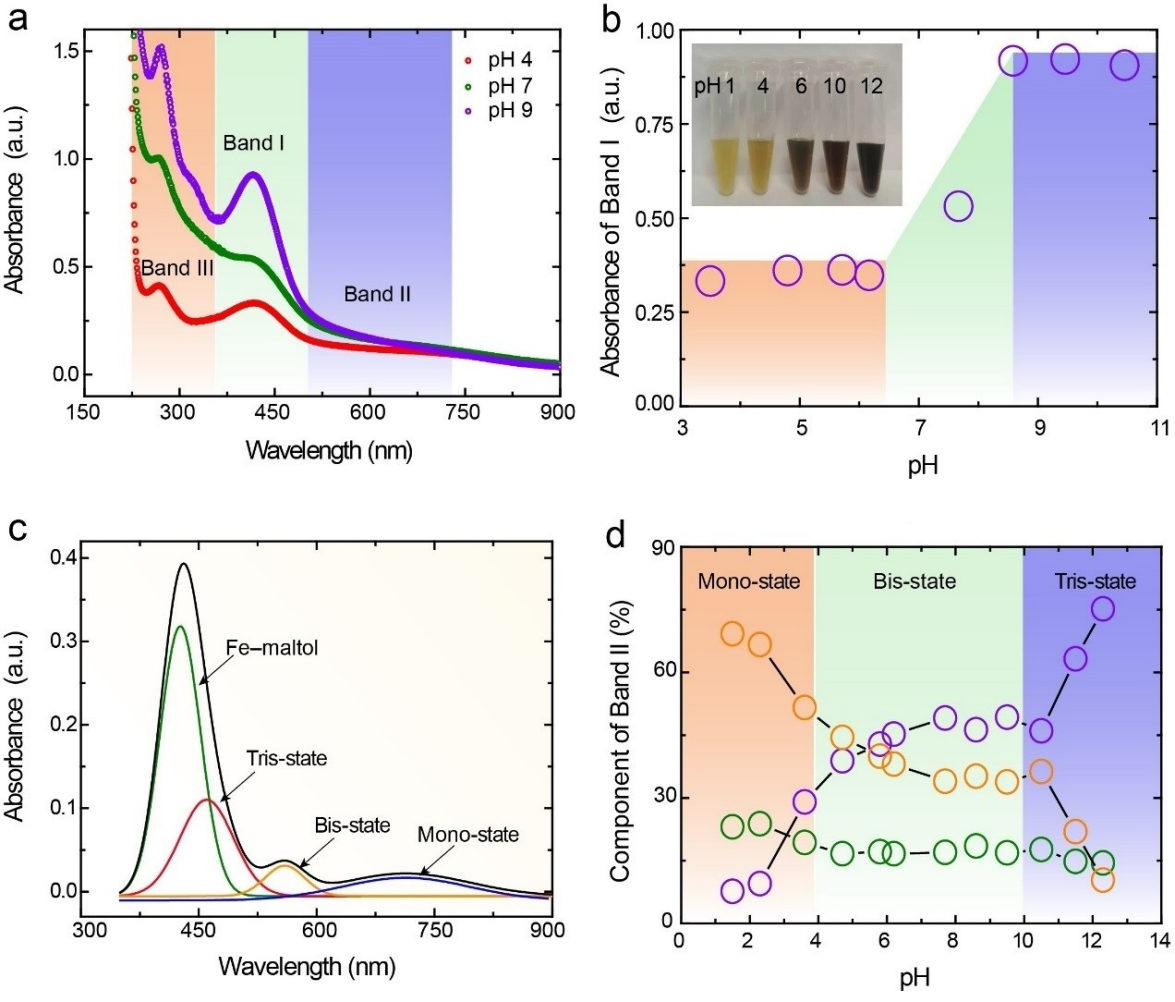
a) UV‐vis spectra of Fe^II^‐QUE complexes at different pH. Band I was attributed to Fe‐maltol coordination, Band II was attributed to Fe‐catechol coordination, and Band III was assigned to π–π* transition in QUE. b) Absorbance values of Fe^II^‐QUE complexes at 420 nm (Band I) at different pH and photograph (inset) of the corresponding Fe^II^‐QUE complex solutions. c) Peaks fitting of Fe^II^‐QUE complexes at pH 9, with the black line representing the cumulative measurement. The fitted peaks include the Fe‐maltol state, and tri‐state, bis‐state, and mono‐state (metal‐catechol coordination). d) Percentage of mono‐, bis‐, and tris‐states, calculated from the area under the curve of Band II, as a function of pH.

To experimentally validate the site selectivity, as revealed by molecular simulations, we investigated the chelation properties of the Fe^II^‐QUE complex at a range of pH values. The UV‐vis spectra of the Fe^II^‐QUE complex showed two ligand‐to‐metal charge‐transfer (LMCT) bands, i.e., a band at about 440 nm (Band I) and a broad band between 500 to 700 nm (Band II), corresponding to metal‐maltol and metal‐catechol coordination, respectively (Figure 2a, Figure S7).^[12]^ Band III was assigned to π–π* transition for QUE. The absorbance of Band I increased from 0.3 to 0.9 with an increase in pH from 3 to 11 (Figure 2b), indicating that the binding affinity of the maltol group is pH‐dependent and favored at higher pH. As the Fe^II^ complex gradually converts into Fe^III^ species upon exposure to air,^[13]^ the formation of the metal‐catechol coordination can be divided into mono‐, bis‐, and tris‐states. The proportion of the mono‐, bis‐, and tris‐coordination species for Band II at different pH (i.e., 1–12) was determined by fitting Gaussian peaks to the UV‐vis spectra curves (Figure 2c, Figure S8). Specifically, the coordination of the mono‐state was the dominant state in acidic environments (e.g., 69 % at pH 2), whereas the tris‐state became the dominant state in alkaline environments (e.g., 75 % at pH 12), and the proportion of bis‐state complexes remained at around 20 % regardless of the pH (Figure 2d, Table S10).

Subsequently, Fe^II^‐QUE complexes were prepared at pH 4, 7, and 9 and analyzed by Fourier transform infrared (FTIR) spectroscopy to identify their coordination modes (Table S11, Figure S9). The C=O stretching at 1668 cm^−1^ for free QUE shifted to 1647 cm^−1^ for Fe^II^‐QUE complexes, implying coordination between the carbonyl oxygen and Fe^II^.^[14]^ The presence of the Fe−O stretching vibration band at 628 cm^−1^ indicated the formation of the coordination complex.^[15]^ Furthermore, the bands at 1443 and 1305 cm^−1^, which were attributed to in‐plane O−H and C−H bending vibrations in the maltol group,^[16]^ were observed in Fe^II^‐QUE complexes at pH 4 (Figure S9 and S10a), while the band at 1474 or 1477 cm^−1^ (i.e., C−O stretching in the catechol group)^[17]^ was observed in Fe^II^‐QUE complexes at pH 7 and pH 9 (Figure S9 and S10b), suggesting that the dominant coordination modes for Fe^II^‐QUE complexes can be readily modulated by altering the pH.

**Figure 3:**
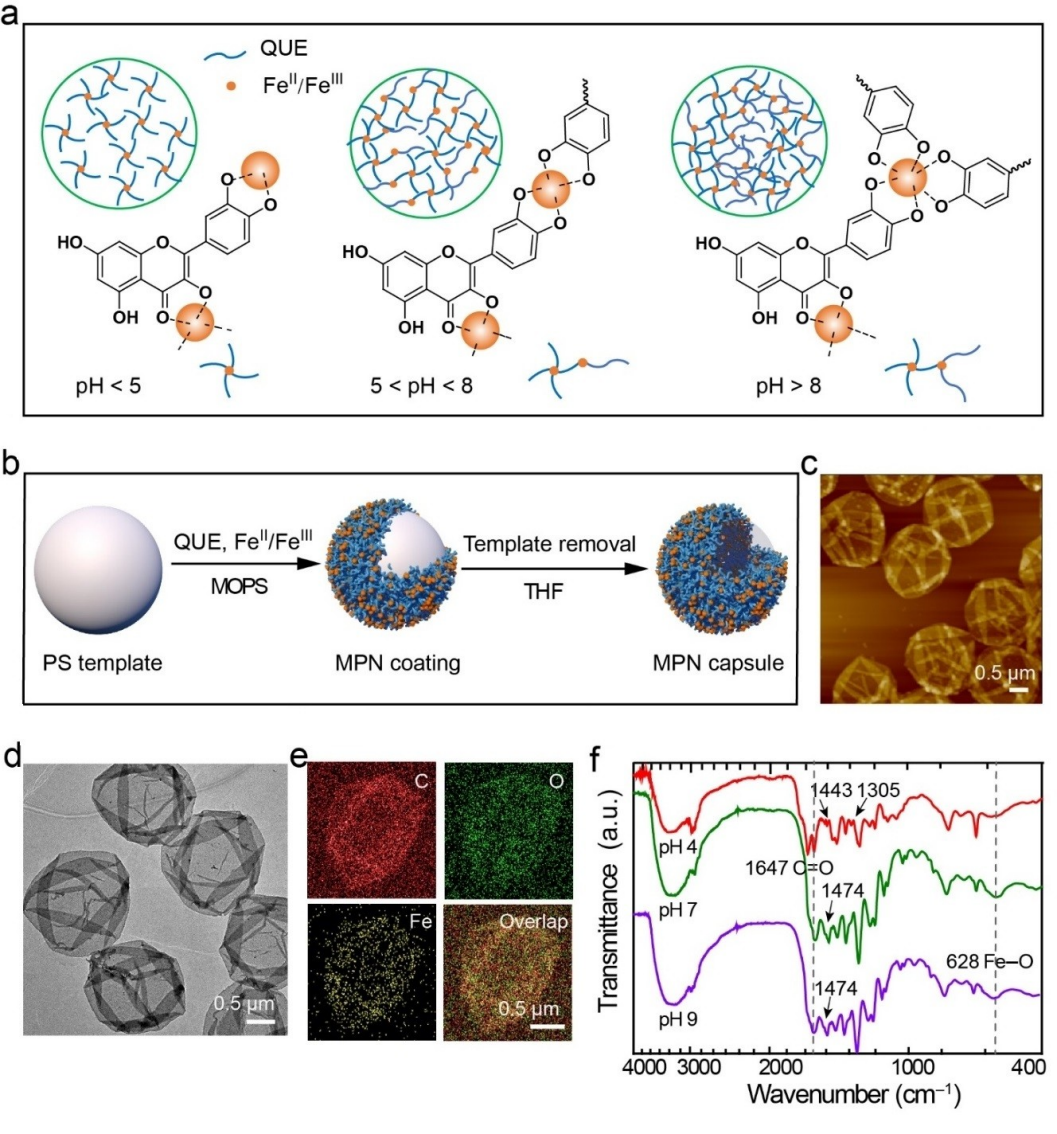
a) Schematic of the coordination interactions between the binding sites of QUE and Fe^II^/Fe^III^ at different pH. At pH<5, dominant complexation occurs with the maltol group. At 5<pH<8, coordination primarily occurs with the catechol group (bis‐state), and at pH>8, coordination primarily occurs with the catechol group (tris‐state). b) Schematic of the coordination‐driven assembly of QUE and Fe^II^/Fe^III^ on sacrificial PS template to form MPN capsules. MOPS, 3‐(*N*‐morpholino)propanesulfonic acid; THF, tetrahydrofuran. c)–e) Morphology of MPN_QUE_ capsules prepared at pH 4, as assessed by AFM (c), TEM (d), and EDX elemental mapping (e). f) FTIR spectra of MPN_QUE_ capsules prepared at different pH.

From our understanding of the chelation states, the selective coordination of Fe^II^‐QUE was explored further to construct conformal coatings on a range of particle substrates with distinct physicochemical properties (e.g., composition, size, and surface chemistry). Specifically, calcium carbonate (CaCO_3_), polystyrene (PS), aminated PS, carboxylated PS, melamine formaldehyde, aminated SiO_2_, poly(methyl methacrylate), and gold nanoparticles were successfully coated as indicated by a shift in the ζ‐potential after coating (Figure S11). To gain mechanistic insight into how the coordination modes regulate the cross‐linking density of the network and interfacial properties, Fe^II^‐QUE MPN capsules (MPN_QUE_ capsules) were engineered at pH 4, 7, or 9 using sacrificial PS particle templates (Figure 3a and b, Figure S12). Transmission electron microscopy (TEM) revealed collapsed capsules with folds and creases after drying (Figure 3d). Energy‐dispersive X‐ray (EDX) spectroscopy mapping showed the presence of elements C, O, and Fe within the MPN_QUE_ capsules (Figure 3e). The ratio between QUE and Fe^II^ in the MPN_QUE_ capsules was dependent on the fabrication conditions (Figure S13, Table S12). Likewise, the single‐wall thickness of the air‐dried capsules was dependent on the fabrication conditions and varied from 8 to 20 nm (Figure 3c, Figure S14), as determined by atomic force microscopy (AFM). Metal coordination within the MPN_QUE_ capsules is further supported by the presence of the Fe−O stretching vibration band in the FTIR spectra of the capsules in Figure 3f. The spectra signals at 625 and 1647 cm^−1^ were ascribed to Fe−O stretching and aryl ketonic stretching (C=O), respectively. The vibrations with frequency at 1443 and 1305 cm^−1^ were assigned to ring deformation with a strong contribution of C−H and O−H bending. The signals at 1474 and 1477 cm^−1^ were attributed to C−C stretching in the phenolic groups. Specifically, the Fe−O band of the MPN_QUE_ capsules could be attributed to two distinct Fe−O bonds including bonding with the carbonyl oxygen of the maltol group (C=O→Fe)^[18]^ and the catechol group (Ph−O→Fe) (Figure S15).^[19]^ The proportion of maltol‐ and catechol‐based coordination in the MPN_QUE_ capsules prepared at different pH was calculated by fitting the Fe−O band of the FTIR spectra curves (Table S13). At pH 4, the maltol group contributed to ≈60 mol % of the coordination, whereas at pH 7 and 9, the maltol and catechol groups showed comparable contributions to the coordination mode within the MPN_QUE_ capsules. In addition, the proportion of mono‐, bis‐, and tris‐states for catechol coordination was examined and the dominant coordination mode for capsules fabricated at pH 4, 7, and 9 was the mono‐state (e.g., 51 mol %), bis‐state (e.g., 45 mol %), and tris‐state (e.g., 60 mol %), respectively (Figure S16, Table S14).

We then investigated the stabilizing mechanisms of MPN_QUE_ capsules by incubating the capsules in different organic solvents and buffers. The MPN_QUE_ capsules remained stable in solutions of 100 mM urea, Tween 20, sodium chloride, and tetrahydrofuran but effectively disassembled in 100 mM of ethylenediaminetetraacetic acid (EDTA) (Figure S17), suggesting that the assemblies are primarily stabilized by coordination interactions rather than covalent, hydrophobic, or π interaction.^[20]^ Moreover, after disassembling MPN_QUE_ capsules by EDTA, the obtained QUE exhibited the same retention time in high‐performance liquid chromatography patterns and similar NMR peaks to pristine QUE, further confirming that QUE did not undergo significant oxidative polymerization during network formation (Figures S18 and S19). The pH‐responsive degradability of the capsules further indicated the coordination‐based nature of the MPNs (Figure S20). Only 3 % of the capsules prepared at pH 7 and pH 9 disassembled after incubation in pH 5 buffer for 3 h, whereas 54 % of the capsules prepared at pH 4 disassembled under the same conditions. This result suggests that an increased proportion of catechol‐based coordination could largely increase the stability of MPN_QUE_ capsules in acidic environments.

**Figure 4:**
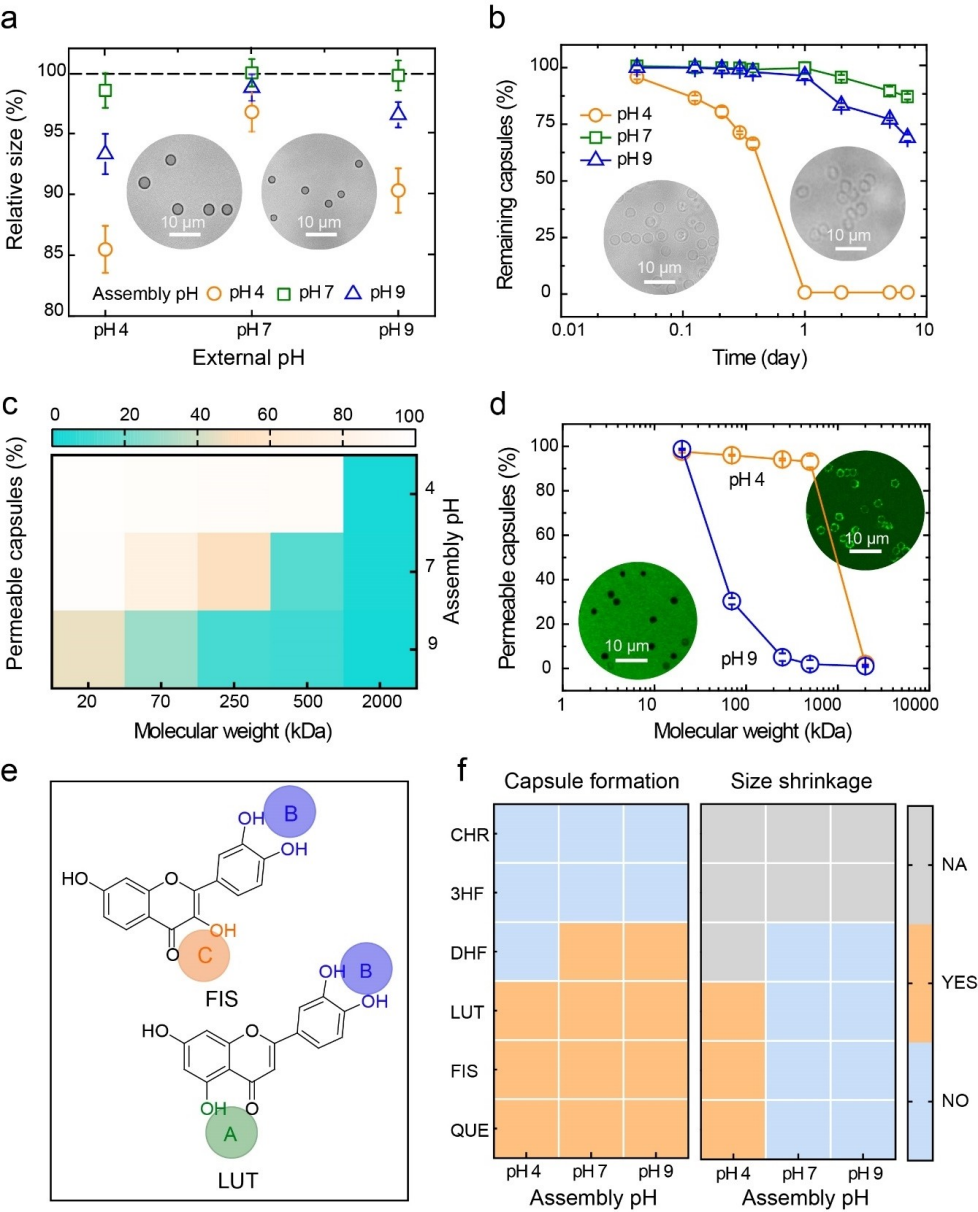
a) Relative size of MPN_QUE_ capsules assembled at different pH (4, 7, and 9) when dispersed in different pH‐buffered solutions. The dotted line represents the template size (3.2 μm normalized to 100 %). Differential interference contrast images (inset) show the capsules at the dispersion pH 7 (left) and pH 4 (right). b) Stability of MPN_QUE_ capsules prepared at pH 4, 7, and 9 in DPBS (pH 7.4) over time. Data are shown as the mean±standard deviation (*n*=3). c) Heat map showing the percentage of MPN_QUE_ capsules (assembled at different pHs) permeable to FITC‐dextran of varying *M*_w_ of 20–2000 kDa. d) Permeability of MPN_QUE_ capsules (assembled at pH 4) measured in pH 4 and pH 9 FITC‐dextran solutions. Inset shows representative confocal laser scanning microscopy images of the corresponding capsules. e) Chemical structures of fisetin (FIS) and luteolin (LUT). The green, orange, and violet circles represent acetylacetone‐, maltol‐, and catechol‐based coordination sites. f) Heat map showing the formation of flavonoid‐based MPN capsules at different pH (left) and size shrinkage of the corresponding capsules upon incubation in 100 mM pH 4 3‐(*N*‐morpholino)propanesulfonic acid (right). NA, not applicable.

Metal‐coordination bonds can break and reform network configurations in response to external factors, thereby enabling dynamic and tunable properties.^[21]^ We therefore investigated how the physicochemical properties of MPN_QUE_ capsules evolve under different conditions. For example, the MPN_QUE_ capsules assembled at pH 4 shrunk to 2.5±0.1 μm (15 % shrinkage relative to PS templates) when dispersed in pH 4 buffer, whereas the capsules fabricated at pH 7 or pH 9 showed negligible size shrinkage (i.e., 2 % and 7 %, respectively) when dispersed in pH 4 buffer (Figure 4a). The shell network of the capsules fabricated at the lower pH of 4 was predominantly constructed via Fe‐maltol and mono‐based Fe‐catechol coordination, and therefore had lower cross‐linking density. The key role of metal‐maltol in the network formation and capsule shrinkage properties were further studied (Figure S21). Specifically, metal‐maltol networks with different metal ions or ligands containing only maltol groups (i.e., 1,4‐dihydroxyanthraquinone and 5,8‐dihydroxy‐1,4‐naphthoquinone) were selected and assembled on PS particles. The intact capsules obtained upon template removal (Figures S22 and S23) indicated the versatility of this site‐selective coordination assembly and that metal‐maltol coordination is significant for stabilizing the capsule structure. Moreover, the Fe^II^‐TA MPN capsules without metal‐maltol coordination exhibited no significant size shrinkage when dispersed in pH 4 buffer (Figure S24). Collectively, these results suggest that the existence of metal‐maltol coordination is essential for the site‐selective coordination of MPN capsules that exhibit size shrinkage properties.

When the assembly pH was increased to 7 and 9, the shell network of the resultant capsules primarily consisted of bis‐ and tris‐based Fe‐catechol coordination, respectively, (Figure 3a, Figure S16d, Table S14) with higher cross‐linking density and greater stability.^[22]^ These observations were consistent with the stability test results of the MPN_QUE_ capsules, where the capsules prepared at pH 7 and pH 9 remained stable in Dulbecco's phosphate‐buffered saline (DPBS, pH 7.4) over 7 days, whereas capsules prepared at pH 4 completely disassembled within a day (Figure 4b). Furthermore, capsules fabricated at pH 4 showed higher permeability than those fabricated at pH 7 and pH 9 (Figure 4c, Figures S25 and S26). For example, 93 % of Fe^III^‐QUE MPN capsules prepared at pH 4 were permeable to fluorescein isothiocyanate (FITC)‐dextran with a molecular weight (*M*_w_) of 500 kDa whereas only 10 % of the capsules prepared at pH 9 were permeable (Figure S26). Such findings are distinct from the previously reported Fe^III^‐TA MPN capsules that displayed higher permeability at higher assembly pH (e.g., pH 9).^[5b]^ A possible reason for this difference could be that the presence of Fe‐maltol coordination leads to closer internal molecular packing in the networks. Notably, the MPN_QUE_ capsules fabricated at pH 4 were highly permeable to 500 kDa FITC‐dextran when the environmental pH was 4 but became almost impermeable to 500 kDa FITC‐dextran when the environmental pH was changed to 9 (Figure 4d), indicating that the microstructure of the network assembled at pH 4 could be further modulated by the environmental pH. Furthermore, the cytotoxicity of the MPN_QUE_ capsules was negligible even at a capsule‐to‐cell ratio of 2000 : 1 (Figure S27), suggesting that these capsules could be used in biotechnological applications.

The versatility of the site‐selective coordination and subsequently tunable physiochemical properties were further investigated using flavonoids with one or two coordination sites. A series of control flavonoids comprising single binding sites (i.e., 3HF, CHR, and DHF) were deposited on PS templates, and only Fe^II^‐DHF assembled at pH 7 and pH 9 could yield free‐standing films after template removal (Figure 4f, Figures S28 and S29a). As DHF possesses one coordination site (i.e., catechol group) and the Fe^II^‐DHF assembly is based on metal‐catechol coordination, the MPN_DHF_ capsules (i.e., fabricated with Fe^II^ and DHF) showed negligible size shrinkage and displayed high permeability when the assembly pH was high (Figure S29b, c). The flavonoids fisetin (FIS) and luteolin (LUT) each have two coordination sites (Figure 4e), suggesting that site‐selective coordination might occur in the Fe^II^‐FIS and Fe^II^‐LUT systems. The MPN_FIS_ capsules were engineered at pH 4, 7, or 9 (Figure S30a) and only those prepared at pH 4 shrunk when exposed to external pH 4 buffer (e.g., 13 % shrinkage relative to PS templates) (Figure S30b). In addition, MPN_FIS_ capsules prepared at pH 4 showed higher permeability than those fabricated at pH 7 (Figure S30c). For example, 98 % of MPN_FIS_ capsules prepared at pH 4 were permeable to FITC‐dextran with a *M*_w_ of 500 kDa, whereas only 2 % of capsules prepared at pH 7 were permeable under the same condition. Similar results were obtained for the MPN_LUT_ capsules (Figure S31). These observations suggest that the present site‐selective strategy could be applied to bidentate or multidentate ligands to construct dynamic networks with tunable properties.

## Conclusion

In summary, we have demonstrated the site‐selective coordination for the dynamic assembly of metal‐phenolic materials. The thermodynamic mechanism of selective binding between metal ions and different chelation groups (i.e., catechol, carbonyl, and hydroxyl groups) within flavonoids was revealed by calculation of the binding energies and p*K*_a_ values, which provides a theoretical framework for regulating the coordination modes in metal‐organic materials. The dominant coordination mode in Fe‐flavonoid was regulated from metal‐catechol to metal‐maltol coordination by tuning the assembly pH, leading to MPN capsules with distinct disassembly kinetics, selective permeability, and size changes. Considering the wide availability of metal ions and multimodal ligands, this site‐selective coordination approach is expected to provide a new pathway to optimizing the properties of metal‐organic materials for emerging applications.

## Conflict of interest

The authors declare no conflict of interest.

1

## Supporting information

As a service to our authors and readers, this journal provides supporting information supplied by the authors. Such materials are peer reviewed and may be re‐organized for online delivery, but are not copy‐edited or typeset. Technical support issues arising from supporting information (other than missing files) should be addressed to the authors.

Supporting Information

## Data Availability

The data that support the findings of this study are available from the corresponding author upon reasonable request.
