# Supplementary material for: Site‐Selective Coordination Assembly of Dynamic Metal‐Phenolic Networks
Source: Angew Chem Int Ed Engl. 2022 Jul 13;61(34):e202208037. doi: 10.1002/anie.202208037 (PMC9546505; doi:10.1002/anie.202208037)
Supplement: Supplementary file 1 — Supporting Information [file ANIE-61-0-s001.pdf]

Angewandte Chemie
Eine Zeitschrift der Gesellschaft Deutscher Chemiker

Supporting Information

Site-Selective Coordination Assembly of Dynamic Metal-Phenolic Networks

*W. Xu, S. Pan, B. B. Noble, J. Chen, Z. Lin, Y. Han, J. Zhou, J. J. Richardson, I. Yarovsky, F. Caruso**

Table of Contents

Table of Contents S2
Section S1. Experimental Procedures S3
Section S1.1 Materials S3
Section S1.2 Characterization S3
Section S1.3 Quantum Mechanical (QM) Methodology S3
Section S1.4 Calculation of pK_a Values and Binding Constants S4
Section S1.5 Determination of Coordination Sites S6
Section S1.6 Fabrication of MPN Capsules from Particle Templates S6
Section S1.7 Quantification of Iron and QUE in MPN Capsules S6
Section S1.8 Stability Experiments S6
Section S1.9 Modification of Microscopy Glass Substrates S6
Section S1.10 Permeability Experiments S7
Section S1.11 Dynamic Size Measurements S7
Section S1.12 Cytotoxicity Assay of MPN Capsules S7
Section S1.13 Minimum Information Reporting in Bio–Nano Experimental Literature (MIRIBEL) S7
Section S2. Supplementary Figures S8
Section S3. Supplementary Tables S22
Section S4. Additional Information S30
Section S5. References S34
Section S6. Author Contributions S34

Section S1. Experimental Procedures

Section S1.1 Materials

Quercetin (QUE), 3-hydroxyflavone (3HF), chrysin (CHR) and 3′,4′-dihydroxyflavone (DHF), luteolin (LUT), fisetin (FIS), 1,4-dihydroxyanthraquinone (DHAQ), 5,8-dihydroxy-1,4-naphthoquinone (DHNQ), tannic acid (TA), zirconium(IV) chloride ($ZrCl_4$), aluminum(III) chloride hexahydrate ($AlCl_3 \cdot 6H_2O$), iron(II) chloride tetrahydrate ($FeCl_2 \cdot 4H_2O$), iron(III) chloride hexahydrate ($FeCl_3 \cdot 6H_2O$), 3-(*N*-morpholino)propanesulfonic acid (MOPS), sodium chloride (NaCl), sodium acetate, fluorescein isothiocyanate (FITC), FITC–dextran of varying average molecular weights (20, 70, 250, 500, and 2000 kDa), and (3-aminopropyl)triethoxysilane (APTES), ethylenediaminetetraacetic acid (EDTA), urea, and Tween 20 were purchased from Sigma-Aldrich (USA). Polystyrene (PS) particles (D = 3.20 ± 0.13 μm, 10 w/v% aq. suspension), carboxyl-modified polystyrene (PS-COOH) (D = 1.86 ± 0.08 μm), and amine-modified polystyrene (PS-NH_2) (D = 1.86 ± 0.08 μm) particles, poly(methyl methacrylate) (PMMA) particles (D = 3.69 ± 0.08 μm), gold nanoparticles (AuNP) (D = 20 nm), aminated silica (SiO_2-NH_2) particles (D = 0.46 ± 0.02 μm), and melamine formaldehyde (MF) particles (D = 2.98 ± 0.06 μm) were purchased from microParticles GmbH. Tetrahydrofuran (THF), 1,4-dioxane, methanol, and ethanol were purchased from Chem-Supply. 2,3-Bis[2-methoxy-4-nitro-5-sulfophenyl]-2*H*-tetrazolium-5-carboxyanilide inner salt (XTT), Dulbecco’s modified eagle medium (DMEM), and Dulbecco’s phosphate-buffered saline (DPBS) were obtained from Life Technologies. All chemicals were used as received without further purification. Milli-Q water with a resistivity of greater than 18.2 MΩ cm was obtained from a three-stage Millipore Milli-Q plus 185 purification system (Millipore Corporation, USA).

Section S1.2 Characterization

UV–vis absorption spectra were recorded on a Specord 250 Plus spectrophotometer (Analytik Jena AG). Fourier transform infrared (FTIR) spectroscopy analysis was conducted on a Tensor–II–FTIR spectrometer. Differential interference contrast (DIC) microscopy images of capsules were taken with an inverted Olympus IX71 microscope. Scanning electron microscopy (SEM) images were obtained using an FEI Quanta 200 field-emission scanning electron microscope, operating at an accelerating voltage of 10 kV. For the SEM experiments, dried samples were coated with Au by using K575X Turbo Sputter Coater. Transmission electron microscopy (TEM) and energy-dispersive X-ray mapping analysis of capsules were performed on an FEI Tecnai TF20 instrument (USA) at an operating voltage of 200 kV. Atomic force microscopy (AFM) experiments were conducted using a JPK NanoWizard II BioAFM instrument. Confocal laser scanning microscopy (CLSM) images were taken with a Nikon A1R+ laser scanning confocal microscope (Nikon Corporation, Japan). ζ-Potential measurements were performed using a Malvern Zetasizer Nano ZS instrument (Malvern Instrument, UK). The metal content in the capsules was determined by inductively coupled plasma optical emission spectrometry (ICP-OES) using an ICP Varian 710-ES instrument. QUE, Fe ion, EDTA, and Fe^{II}–QUE metal–phenolic network (MPN) components after degradation of the MPN_{QUE} capsules by EDTA were purified using high-performance liquid chromatography (HPLC) equipped with a UV detector (Shimadzu, Kyoto, Japan). HPLC analysis was performed on a C18 column (4.6 mm internal diameter (ID) using gradient mode with water containing 0.1% formic acid and acetonitrile containing 0.1% formic acid. The flow rates, operating temperature, and UV wavelength for detection were set at 1 mL min^{-1}, 35 °C, and 254 nm, respectively.The chemical structures of free QUE and QUE after MPN assembly procedure were determined by proton nuclear magnetic resonance (^1H NMR) spectroscopy on a 400 MHz NMR spectrometer (JEOL Ltd., Akishima, Tokyo, Japan).

Section S1.3 Quantum Mechanical (QM) Methodology

All density functional theory (DFT) and wavefunction calculations were performed in ORCA 5.0.1.[1] Geometry optimizations were performed using the B97M-V functional[2] and def2-TZVP basis set,[3] using the resolution of the identity (RIJ) approximation and a def2/J auxiliary basis set.[4] These optimizations were performed directly in a conductor-like polarizable continuum model (CPCM) solvent field,[5] with water as the solvent. Improved energies were calculated with DLPNO-CCSD(T)[6] and the cc-pVTZ basis set,[7] using the RIJCOSX approximation and the cc-pVTZ/C and def2/J auxiliary basis sets.[4,8] These DLPNO-CCSD(T) single point calculations were also performed in a CPCM solvent field. For calculations involving anionic Fe(III) complexes (and their conjugate acids), DLPNO-CCSD(T)/aug-cc-pVTZ calculations were performed, using an aug-cc-pVTZ/C auxiliary basis set. Due to linear dependency issues, the RIJCOSX approximation was not used for these calculations. All DFT and DLPNO-CCSD(T) calculations were performed using ‘Tight’ Self-Consistent Field (SCF) cut-offs.

Having optimized the structures, (numerical) frequencies were calculated in the CPCM solution to confirm structures were true minima, possessing no imaginary frequencies. Solution-phase Gibbs free energies were then calculated using the “direct method”.[9] For systems that undergo large geometry changes upon solvation, the direct method affords energetics that are superior to those obtained using (gas phase to solution) thermocycles.[9] Given the high partial charges on atoms within these metal complexes, significant geometry changes upon solvation (in water) would be anticipated. The default standard state used within ORCA 5.0.1. for entropic components (even in a CPCM solvent field) is based on the statistical mechanics for an ideal gas (evaluated at 1 atm of pressure and 25 °C). Thus, appropriate standard state corrections were applied to ensure all binding energies and pK_a values were calculated at a standard state for solutes in solution (of 1 mol L^{-1} and 25 °C). This correction takes the following form.

$$\Delta G^{\mathrm{1atm}\rightarrow\mathrm{1M}} = \Delta m RT \ln\left[\frac{RT}{P}\right] \quad (\mathrm{Eq.\,1})$$

Here Δm is the change in moles upon reaction, R is the ideal gas constant (8.3145 J mol^{-1} K^{-1}), T is the temperature of interest at which the Gibbs free energy is also evaluated (typically 298.15 K) and P is pressure (101.325 kPa = 1 atm).

For reactions that either generate or consume water (i.e., reactions where water is a product or reagent), a further state correction is required. The standard state for any liquid is the pure substance at 1 atm of pressure, so the standard state of liquid water should be [H_2O] = 55.5 mol L^{-1} (rather than 1 mol L^{-1} for an aqueous solute). Gibbs free energies for reactions involving water as a reagent must be further corrected by adding the following additional term:

$$\Delta G^{\mathrm{1M}\rightarrow\mathrm{55.5M}} = -nRT\ln[\mathrm{H_2O}] \quad (\mathrm{Eq.\,2})$$

Here n is the number of moles of water acting as a reagent. Conversely, for reactions that generate water as a product, Gibbs free energies are corrected by the following term:

$$\Delta G^{1\mathrm{M}\rightarrow 55.5\mathrm{M}} = +nRT\ln[\mathrm{H_2O}] \qquad \text{(Eq. 3)}$$

Here n is the number of moles of water produced. We should emphasize these standard state corrections are only required for reaction energies calculated via QM approaches.

For QUE and DHF systems, both syn- and anti-conformations of the free ligand and respective Fe^{II} and Fe^{III} complexes were considered for all protonation states. Theoretically, any of the unique aqua ligands can undergo deprotonation, with this process forming distinct coordination isomers (e.g., with OH groups in either axial or equatorial positions). Thus, deprotonation of all unique aqua ligands was considered to identify the most energetically favorable coordination geometry for a given overall protonation state. Similarly, for DHF and QUE complexes (with Site B binding), deprotonation of the Fe-bound para- and meta-OH group was considered, as these processes lead to different coordination isomers. In all cases, reported energetics are based on the lowest energy conformation identified. All Fe^{II} and Fe^{III} complexes were modeled in their respective high-spin (quintet and sextet) configurations as test calculations indicated these were significantly more stable than possible low-spin states. Molecular graphics were rendered with UCSF Chimera, developed by the Resource for Biocomputing, Visualization, and Informatics at the University of California, San Francisco, with support from NIH P41-GM103311.37.[10]

Section S1.4 Calculation of pK_a Values and Binding Constants

Within standard continuum solvation models, solvation energies are profoundly influenced by the overall charge of the metal–ligand (ML) complex as well as the coordination number (CN) and oxidation/spin state of the metal ion.[11] Absolute predictions of pK_a values for ML complexes are prone to significant solvation errors because deprotonation decreases the overall charge of the complex and often decreases the CN of the metal ion.[11]

However, solvation errors are largely systematic and can be offset by considering concurrent protonation of a (structurally similar) reference conjugate base.[12] For this purpose, the corresponding Fe^{II} and Fe^{III} aqua hydrolysis constants (Rxns 1 and 2 and Rxns 3–6, respectively) can be used as reference reactions:

$$[\mathrm{Fe^{II}(H_2O)_6}]^{2+}_{(aq)} \longrightarrow [\mathrm{Fe^{II}(H_2O)_5(OH)}]^{+}_{(aq)} + \mathrm{H}^{+}_{(aq)}, \qquad \mathrm{p}K_\mathrm{a} = 9.5 \qquad \text{(Rxn 1)}$$

$$[\mathrm{Fe^{II}(H_2O)_5(OH)}]^{+}_{(aq)} \longrightarrow [\mathrm{Fe^{II}(H_2O)_3(OH)_2}]^{0}_{(aq)} + \mathrm{H}^{+}_{(aq)} + \mathrm{H_2O_{(l)}}, \qquad \mathrm{p}K_\mathrm{a} = 11.1 \qquad \text{(Rxn 2)}$$

$$[\mathrm{Fe^{III}(H_2O)_6}]^{3+}_{(aq)} \longrightarrow [\mathrm{Fe^{III}(H_2O)_5(OH)}]^{2+}_{(aq)} + \mathrm{H}^{+}_{(aq)}, \qquad \mathrm{p}K_\mathrm{a} = 2.2 \qquad \text{(Rxn 3)}$$

$$[\mathrm{Fe^{III}(H_2O)_5(OH)}]^{2+}_{(aq)} \longrightarrow [\mathrm{Fe^{III}(H_2O)_4(OH)_2}]^{+}_{(aq)} + \mathrm{H}^{+}_{(aq)}, \qquad \mathrm{p}K_\mathrm{a} = 3.5 \qquad \text{(Rxn 4)}$$

$$[\mathrm{Fe^{III}(H_2O)_4(OH)_2}]^{+}_{(aq)} \longrightarrow [\mathrm{Fe^{III}(H_2O)_2(OH)_3}]^{0}_{(aq)} + \mathrm{H}^{+}_{(aq)} + \mathrm{H_2O_{(l)}}, \qquad \mathrm{p}K_\mathrm{a} = 6.3 \qquad \text{(Rxn 5)}$$

$$[\mathrm{Fe^{III}(H_2O)_2(OH)_3}]^{0}_{(aq)} \longrightarrow [\mathrm{Fe^{III}(OH)_4}]^{-}_{(aq)} + \mathrm{H}^{+}_{(aq)} + \mathrm{H_2O_{(l)}}, \qquad \mathrm{p}K_\mathrm{a} = 9.6 \qquad \text{(Rxn 6)}$$

As these hydrolysis reactions (Rxns 1–6) are important for describing the acid/base characteristics of iron species in aqueous environments, the reference pK_a values are known experimentally with relatively high precision.

Speciation of these Fe^{III} aqua complexes in different protonation states has been determined in previous work (see Ref [13]). The corresponding reference experimental pK_a values for these Fe^{III} complexes were taken from Ref [13]. However, we should note that there is some experimental variability in these values, particularly for Rxn 5 and Rxn 6. For instance, Ref [14] reports pK_a values for Rxn 5 that are around 2 units higher (8.54) and values for Rxn 6 that are around 2 units lower (7.41) than the corresponding reference experimental pK_a values from Ref [13]. Although the choice of reference values does not impact the site selectivity of the respective complexes directly (see Table S9 below), it does impact the pH range where these complexes predominate (see Figure S1). These reference values are also dependent on ionic strength (I).[14] The reported pK_a values for Rxns 3–6 are 2.9, 3.9, 6.5, and 8.6, respectively, at I = 0.5 mol L^{-1}, and 2.7, 4.3, 5.5, and 8.1, respectively, at I = 1.0 mol L^{-1}.[15] Formally, the QM calculations are performed at I = 0 mol L^{-1}, thus the reference pK_a values taken at I = 0 mol L^{-1} were used for Rxns 1–6. However, the effect of non-zero ionic strength could be roughly approximated by using reference pK_a values at I = 0.5 and 1.0 mol L^{-1} (see Figure S2).

Although the octahedral geometries of $[Fe^{II}(H_2O)_6]^{2+}$ and $[Fe^{II}(H_2O)_5(OH)]^{+}$ are well established (see Ref [16]), the precise structure and speciation of $[Fe^{II}(H_2O)_n(OH)_2]^0$ is more speculative. Experimental identification of this species is difficult because of the poor solubility of Fe^{II} species at high pH and their instability toward aerobic oxidation.[17] However, calculations indicate that the octahedral $[Fe^{II}(H_2O)_4(OH)_2]^0$ complex, the trigonal–bipyramidal $[Fe^{II}(H_2O)_3(OH)_2]^0$ complex, and the tetrahedral $[Fe^{II}(H_2O)_2(OH)_2]^0$ complex all have reasonably comparable stabilities (see Figure S3).

Accounting for the necessary standard state corrections (see Eqs 1–3 above), the trigonal–bipyramidal $[Fe^{II}(H_2O)_3(OH)_2]^0$ complex was found to be the most stable and thus was assumed to be the dominant species in Rxn 2. The corresponding experimental pK_a values for these Fe^{II} aqua species were taken from Ref [17].

For a given ML complex of interest, isodesmic proton-transfer reactions can be easily constructed. For instance, for Fe^{II} and a model diprotic ligand (LH_2) the following isodesmic proton-transfer reactions can be considered:

$$\underbrace{[\mathrm{Fe^{II}(H_2O)_4(LH_2)}]^{2+}_{(aq)}}_{\text{Acid}} + \underbrace{[\mathrm{Fe^{II}(H_2O)_5(OH)}]^{+}_{(aq)}}_{\substack{\text{Reference}\\ \text{Conjugate Base}}} \longrightarrow \underbrace{[\mathrm{Fe^{II}(H_2O)_4(LH)}]^{+}_{(aq)}}_{\text{Conjugate Base}} + \underbrace{[\mathrm{Fe^{II}(H_2O)_6}]^{2+}_{(aq)}}_{\substack{\text{Reference}\\ \text{Acid}}}$$

(Rxn 7)

$$\underbrace{[\mathrm{Fe^{II}(H_2O)_4(LH)}]^+_{(aq)}}_{\text{Acid}} + \underbrace{[\mathrm{Fe^{II}(H_2O)_3(OH)_2}]^0_{(aq)}}_{\substack{\text{Reference}\\ \text{Conjugate Base}}} \longrightarrow \underbrace{[\mathrm{Fe^{II}(H_2O)_3(L)}]^0_{(aq)}}_{\text{Conjugate Base}} + \underbrace{[\mathrm{Fe^{II}(H_2O)_5(OH)}]^+_{(aq)}}_{\substack{\text{Reference}\\ \text{Acid}}} \quad \text{(Rxn 8)}$$

Rxns 7 and 8 compensate for the decrease in the overall charge of a given ML complex (upon deprotonation) via a corresponding increase to the overall charge of a reference metal–aqua complex (through protonation). These metal–aqua complexes are chosen so that the reference acid has the same overall charge as the metal–ligand complex in its protonated form. These proton-transfer reactions also conserve the oxidation states and total CN of the metal ions.

Relative pK_a values for the ML complexes of interest, with respect to the corresponding metal–aqua complexes, can be determined via the Gibbs free energies for these isodesmic proton-transfer (PT) reactions (ΔG_{PT}):

$$\mathrm{p}K_\mathrm{a}[\mathrm{MLH_2}\ \text{with respect to Rxn 1}] = \frac{\Delta G_\mathrm{PT}[\text{Rxn 7}]}{RT\ln(10)} \quad \text{(Eq. 4)}$$

$$\mathrm{p}K_\mathrm{a}[\mathrm{MLH}\ \text{with respect to Rxn 2}] = \frac{\Delta G_\mathrm{PT}[\text{Rxn 8}]}{RT\ln(10)} \quad \text{(Eq. 5)}$$

The absolute pK_a values can then be determined from these relative pK_a's and the experimentally established values for the reference metal–aqua hydrolysis reactions:

$$\mathrm{p}K_\mathrm{a}[\mathrm{MLH_2}] = \mathrm{p}K_\mathrm{a}[\mathrm{MLH_2}\ \text{with respect to Rxn 1}] + \mathrm{p}K_\mathrm{a}[\text{Rxn 1}] \quad \text{(Eq. 6)}$$

$$\mathrm{p}K_\mathrm{a}[\mathrm{MLH}] = \mathrm{p}K_\mathrm{a}[\mathrm{MLH}\ \text{with respect to Rxn 2}] + \mathrm{p}K_\mathrm{a}[\text{Rxn 2}] \quad \text{(Eq. 7)}$$

Whereas most of these proton-transfer reactions are isodesmic, those involving reference Rxn 6 are not because water is required as a reagent to balance the reaction. For instance, for a model diprotic ligand (LH_2), the proton-transfer reaction is:

$$\underbrace{[\mathrm{Fe^{III}(H_2O)_2(OH)(L)}]^0_{(aq)}}_{\text{Acid}} + \underbrace{[\mathrm{Fe^{III}(OH)_4}]^-_{(aq)}}_{\substack{\text{Reference}\\ \text{Conjugate Base}}} + \mathrm{H_2O_{(l)}} \longrightarrow \underbrace{[\mathrm{Fe^{III}(H_2O)(OH)_2(L)}]^-_{(aq)}}_{\text{Conjugate Base}} + \underbrace{[\mathrm{Fe^{III}(H_2O)_2(OH)_3}]^0_{(aq)}}_{\substack{\text{Reference}\\ \text{Acid}}} \quad \text{(Rxn 9)}$$

For reactions of the form of Rxn 9, the QM-calculated Gibbs free energies must be corrected according to Eq. 2 to account for the consumption of water.

The Gibbs free energies for complexation of a neutral (fully protonated) ligand to a metal ion, $\Delta G_\mathrm{B}[\mathrm{LH_2}]$, can be determined. For instance, for FeII and a model diprotic ligand (LH2) the following reaction describes initial complexation:

$$\underbrace{[\mathrm{Fe^{II}(H_2O)_6}]^{2+}_{(aq)}}_{\text{Metal–aqua complex}} + \underbrace{\mathrm{LH_2}_{(aq)}}_{\text{free ligand}} \longrightarrow \underbrace{[\mathrm{Fe^{II}(H_2O)_4(LH_2)}]^{2+}_{(aq)}}_{\substack{\text{Metal–ligand complex}\\ \text{fully protonated}}} + \underbrace{2\mathrm{H_2O_{(l)}}}_{\text{free water}} \quad \text{(Rxn 10)}$$

This ligand-transfer reaction does not have a significant solvation component, as the reactant and product metal complexes have the same overall charge, CN, and oxidation state. That is, Rxn 10 would not be particularly sensitive to the use of a continuum solvation model. The QM-calculated Gibbs free energies for Rxn 10 must be corrected (via Eq. 3) to account for the concentration of bulk liquid water (55.5 mol L^{-1}).

Having determined the binding affinities for the fully protonated ligands and the pK_a values for the respective ML complexes, other energetic terms can be determined via Hess's law thermocycles. For instance, ligand binding and spontaneous deprotonation(s) of the resulting metal–ligand complex(es) can be described by the following reactions:

$$\underbrace{[\mathrm{Fe^{II}(H_2O)_6}]^{2+}_{(aq)}}_{\text{Metal–aqua complex}} + \underbrace{\mathrm{LH_2}_{(aq)}}_{\text{free ligand}} \longrightarrow \underbrace{[\mathrm{Fe^{II}(H_2O)_4(LH)}]^{+}_{(aq)}}_{\substack{\text{Metal–ligand complex}\\ \text{partially deprotonated}}} + \underbrace{2\mathrm{H_2O_{(l)}}}_{\text{free water}} + \underbrace{\mathrm{H^+_{(aq)}}}_{\text{free proton}} \quad \text{(Rxn 11)}$$

$$\underbrace{[\mathrm{Fe^{II}(H_2O)_6}]^{2+}_{(aq)}}_{\text{Metal–aqua complex}} + \underbrace{\mathrm{LH_2}_{(aq)}}_{\text{free ligand}} \longrightarrow \underbrace{[\mathrm{Fe^{II}(H_2O)_3(L)}]^{0}_{(aq)}}_{\substack{\text{Metal–ligand complex}\\ \text{fully deprotonated}}} + \underbrace{3\mathrm{H_2O_{(l)}}}_{\text{free water}} + \underbrace{2\mathrm{H^+_{(aq)}}}_{\text{free protons}} \quad \text{(Rxn 12)}$$

Direct calculations of Rxns 11 and 12 would be prone to significant solvation errors arising from the decreased charge on the metal–ligand complexes, changes to the CN of the metal ion, and generation of free proton(s). However, the energetics of these processes (Rxns 11 and 12) can be expressed, via a Hess's law thermocycle, as the sum of the corresponding energetics for neutral ligand binding and deprotonation(s) of the resulting metal–ligand complex(es):

$$\Delta G^*_\mathrm{B}[\mathrm{MLH}] = \Delta G^\mathrm{corr}_\mathrm{B}[\mathrm{LH_2}] + \Delta G_\mathrm{A}[\mathrm{MLH_2}] \quad \text{(Eq. 8)}$$

$$\Delta G^*_\mathrm{B}[\mathrm{ML}] = \Delta G^\mathrm{corr}_\mathrm{B}[\mathrm{LH_2}] + \Delta G_\mathrm{A}[\mathrm{MLH_2}] + \Delta G_\mathrm{A}[\mathrm{MLH}] \quad \text{(Eq. 9)}$$

The major advantage of using a relative pKa approach is that it corrects systematic errors in the metal–ligand deprotonation components, $\Delta G_\mathrm{A}[\mathrm{MLH_2}]$ and $\Delta G_\mathrm{A}[\mathrm{MLH}]$, caused by the continuum solvation model. These systematic errors would otherwise adversely affect the accuracy of $\Delta G^*_\mathrm{B}[\mathrm{MLH}]$ and $\Delta G^*_\mathrm{B}[\mathrm{ML}]$. These Gibbs free energies describe binding at pH = 0, (i.e., [H+] = 1.0 mol L^{-1}) but can be easily adjusted for other pH values.

Having determined $\Delta G^*_\mathrm{B}[\mathrm{MLH}]$ and $\Delta G^*_\mathrm{B}[\mathrm{ML}]$ accurately, binding of the deprotonated ligand to the metal ion can also be considered:

$$\underbrace{[\mathrm{Fe^{II}(H_2O)_6}]^{2+}_{(\mathrm{aq})}}_{\text{Metal–aqua complex}} + \underbrace{\mathrm{LH^-_{(aq)}}}_{\substack{\text{free ligand}\\ \text{partially deprotonated}}} \longrightarrow \underbrace{[\mathrm{Fe^{II}(H_2O)_4(LH)}]^{+}_{(\mathrm{aq})}}_{\substack{\text{Metal–ligand complex}\\ \text{partially deprotonated}}} + \underbrace{\mathrm{2H_2O_{(l)}}}_{\text{free water}} \qquad \text{(Rxn 13)}$$

$$\underbrace{[\mathrm{Fe^{II}(H_2O)_6}]^{2+}_{(\mathrm{aq})}}_{\text{Metal–aqua complex}} + \underbrace{\mathrm{L^{2-}_{(aq)}}}_{\substack{\text{free ligand}\\ \text{fully deprotonated}}} \longrightarrow \underbrace{[\mathrm{Fe^{II}(H_2O)_3(L)}]^{0}_{(\mathrm{aq})}}_{\substack{\text{Metal–ligand complex}\\ \text{fully deprotonated}}} + \underbrace{\mathrm{3H_2O_{(l)}}}_{\text{free water}} \qquad \text{(Rxn 14)}$$

The energetics for Rxns 13 and 14 can be determined using similar Hess's Law thermocycles and the pKa values of the free ligands:

$$\Delta G_{\mathrm{B}}[\mathrm{LH^-}] = \Delta G^*_{\mathrm{B}}[\mathrm{MLH}] - \Delta G_{\mathrm{A}}[\mathrm{LH_2}] \qquad \text{(Eq. 10)}$$

$$\Delta G_{\mathrm{B}}[\mathrm{L^{2-}}] = \Delta G^*_{\mathrm{B}}[\mathrm{ML}] - \Delta G_{\mathrm{A}}[\mathrm{LH_2}] - \Delta G_{\mathrm{A}}[\mathrm{LH^-}] \qquad \text{(Eq. 11)}$$

Although many of the pKa values of the free flavonoid ligands are known experimentally, they can also be calculated using a similar relative pKa approach (as described above). Although binding energies could be further corrected to account for the impact of metal–aqua complex hydrolysis reactions and free ligand ionization, neither of these processes impacts on relative site selectivity (though absolute effective binding energies are affected). Given the focus was on relative predictions of site selectivity, neither ligand ionization nor metal–aqua complex hydrolysis was explicitly considered in this work.

Section S1.5 Determination of Coordination Sites

UV–vis spectrophotometry was used to determine the coordination sites of Fe^{II}–flavonoid complexes in aqueous solution. The samples were prepared as follows: solutions of free flavonoids dissolved in methanol to achieve a concentration of 0.25 mM were prepared. The Fe^{II}–QUE complexes were obtained by mixing 0.5 mM $FeCl_2{\cdot}4H_2O$ in water with 0.25 mM QUE in methanol, and the pH of the complex solution was adjusted to within a range of 1–12 by adding 1 mM NaOH or 1 mM HCl solution. To obtain the other Fe^{II}–flavonoid complexes, 0.25 mM 3HF, CHR, or DHF was mixed with 0.5 mM $FeCl_2{\cdot}4H_2O$, and the solution of the complex was adjusted to pH 4, 7, or 9. FTIR spectroscopy was used to determine the coordination between Fe^{II} and flavonoids in the solid state. The samples were prepared as follows: 500 µL of $FeCl_2{\cdot}4H_2O$ (10 mg mL^{-1} in water) and 500 µL of QUE (5 mg mL^{-1} in methanol) were added and vortexed for 10 s, and the pH of the mixed solution was adjusted by adding 1000 µL of 100 mM MOPS (pH 4, 7, or 9). The mixture was kept still for 2 h. The free complexes were removed by centrifugation (2000 *g*, 1 min) and the sediments were washed three times with Milli-Q water. The Fe^{II}–QUE complexes were freeze-dried before the FTIR spectroscopy measurements. Other Fe^{II}–flavonoid complexes were prepared in the same manner as described for the Fe^{II}–QUE complexes. The Gaussian function fitting approach was used to perform curve fitting.

Section S1.6 Fabrication of MPN Capsules from Particle Templates

All flavonoids and metal solutions were prepared freshly for immediate use. The standard protocol used for capsule preparation was as follows: 30 µL of PS particles was washed twice with Milli-Q water (2000 *g*, 1 min) and suspended in 365 µL of water. Then 75 µL of methanol, 13.2 µL of $FeCl_2{\cdot}4H_2O$ (10 mg mL^{-1} in water), and 20 µL of QUE (5 mg mL^{-1} in methanol) were added successively to the PS dispersion at room temperature (25 °C) followed by brief vortexing and sonication. The pH of the mixed dispersion was adjusted by adding 500 µL of 100 mM MOPS (pH 4, 7, or 9) and the suspension was vortexed for 60 s. The mixture was allowed to sit undisturbed for 2 h to achieve sufficient film formation and adherence. Non-coating complexes were removed by centrifugation (2000 *g*, 1 min) and the pellet was washed three times with Milli-Q water. To obtain MPN capsules, 1000 µL of THF was added to dilute the suspension and the particles were incubated with THF for at least 3 h. Then the pellets were washed four times with THF (2000 *g*, 3 min), and the resulting hollow capsules were resuspended in 300 µL of water for characterization. The same protocol was followed for the fabrication of MPN capsules using other particle templates.

For the preparation of MPN coatings with other flavonoids, 40 µL of 2.5 mg mL^{-1} 3HF or LUT, 100 µL of 1 mg mL^{-1} CHR, or 50 µL of 2 mg mL^{-1} DHF or FIS was used, and the same fabrication process was applied. For fabricating MPN coatings at pH 4 using different metal ions and ligands, 15 µL of 5 mg mL^{-1} $ZrCl_4$ or 11 µL of 15 mg mL^{-1} $AlCl_3{\cdot}6H_2O$ and 40 µL of 2 mg mL^{-1} DHNQ, 31 µL of 2 mg mL^{-1} DHAQ, or 56 µL of 10 mg mL^{-1} TA were used, and the same fabrication process was applied.

Section S1.7 Quantification of Iron and QUE in MPN Capsules

ICP-OES and flow cytometry (Apogee A50-Micro flow cytometer) were used to determine the amount of iron in the MPN capsules. First, an iron ICP standard solution was diluted to 0.1, 0.5, 1, 5, and 10 ppm with 5% nitric acid (HNO_3) to construct calibration curves. The number of MPN capsules was determined by flow cytometry. To determine the content of iron, MPN capsules were disassembled in 65% HNO_3 and then diluted with Milli-Q water to 5% HNO_3 for ICP analysis. To quantify the amount of QUE per capsule, a calibration curve of QUE was constructed, and the disassembled capsule solution was analyzed by UV–vis spectrophotometry at an absorption wavelength of 371 nm.

Section S1.8 Stability Experiments

MPN capsule suspensions (1.0×10^7 capsules μL^{-1}) in 100 mM glycine–HCl (pH 3.0), 100 mM sodium acetate (pH 5.0), 100 mM MOPS (pH 7.0), or 100 mM MOPS (pH 9.0) were incubated at room temperature for the desired time. At indicated time points, 10 µL of the capsule suspensions was diluted with 290 µL of water for flow cytometric analysis to count the number of capsules. Capsule counting was carried out with an Apogee A50-Micro flow cytometer (Apogee Flow Systems, UK). Data are shown as the mean ± standard deviation (SD) of three independent measurements. MPN capsules were dissolved in 100 mM urea, Tween 20, NaCl, EDTA (pH 7.4), or THF.

Section S1.9 Modification of Microscopy Glass Substrates

Modified glass substrates were used for the capsule permeability and size measurements. For the modification, a 0.5% APTES solution was prepared by dissolving 400 µL of ATPES in 80 mL of ethanol. Aminated microscopy glass substrates were obtained by immersing the substrates in 0.5% APTES solution for 24 h and subsequent thorough rinsing with ethanol and Milli-Q water. The cleaned substrates were then dried under a stream of air before use.

Section S1.10 Permeability Experiments
To test capsule permeability, 200 μL of an MPN-coated PS suspension was added to the modified microscopy glass substrate and then the substrate was kept still for 10 min. The glass substrate was then immersed in THF to remove the PS templates and rinsed with Milli-Q water. Then, 200 μL of FITC–dextran (1 mg mL^{-1} in solution at the desired pH; M_w = 20, 70, 250, 500, 2000 kDa) was added to the capsule area on the glass substrate and incubated for 10 min. The capsules (n = 100) were examined by CLSM. Capsules with dark interiors were regarded as impermeable, whereas those with interiors of the same fluorescence intensity as the outer environment were considered to be permeable.

Section S1.11 Dynamic Size Measurements
For the dynamic size measurements, 10 μL of an MPN-coated PS suspension was added to the modified microscopy glass substrate and then the substrate was kept still for 5 min. Then, 200 μL of 1,4-dioxane was added in situ to remove the PS templates, and excess 1,4-dioxane was aspirated. Subsequently, 200 μL of 100 mM MOPS (pH 4, 7, or 9) was added to the capsule area on the glass substrate and incubated for 10 min. For measuring the MPN capsule size under the above solutions, the diameter of the capsules (n = 20) was measured by DIC microscopy.

Section S1.12 Cytotoxicity Assay of MPN Capsules
The XTT-based in vitro cytotoxicity assay was performed to assess cell toxicity of the MPN capsules. XTT was dissolved in complete DMEM (with 10% FBS) to form 0.2 mg mL^{-1} solution, and phenazine methosulfate (PMS) was dissolved in DPBS to form 1 mM solution. The XTT reagent was activated by mixing with PMS solution at a volume ratio of 400:1. PC-3 cells or A549 cells were seeded on a 96-well plate at a cell density of 2×10^4 cells per well. After incubation with the capsules at different drug dosages for 48 h, the media in the 96-well plate was aspirated and replaced with 100 μL of fresh activated XTT media. The cells were further incubated for 4 h and the absorbance at 475 nm was measured relative to non-treated cells.

Section S1.13 Minimum Information Reporting in Bio–Nano Experimental Literature (MIRIBEL)
The studies conducted herein, including material characterization, biological characterization, and experimental details, conform to the MIRIBEL reporting standard for bio–nano research,[18] and we include a companion checklist of these components herein.

Section S2. Supplementary Figures

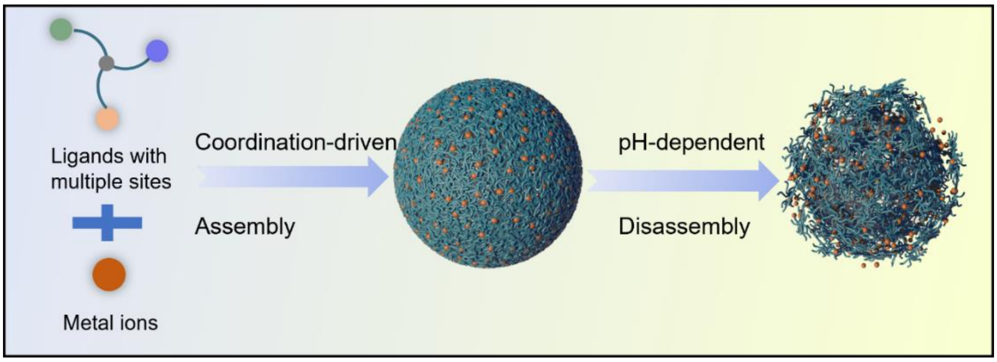

Figure S1. Schematic of the coordination-driven assembly and pH-dependent disassembly between organic ligands with multiple chelation sites and metal ions.

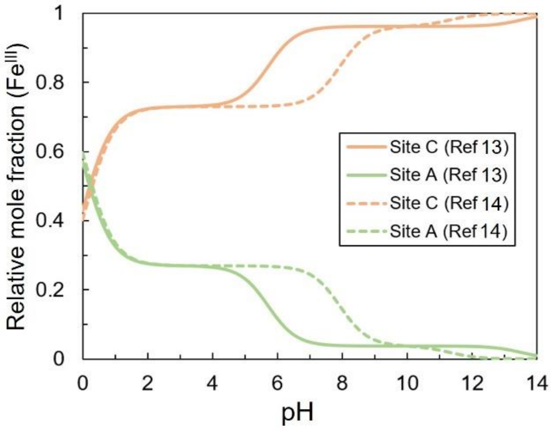

Figure S2. Comparison of the predicted QUE site selectivity for Fe^{III} as a function of pH using reference hydrolysis data from Ref [13] (solid lines) and Ref [14] (dashed lines).

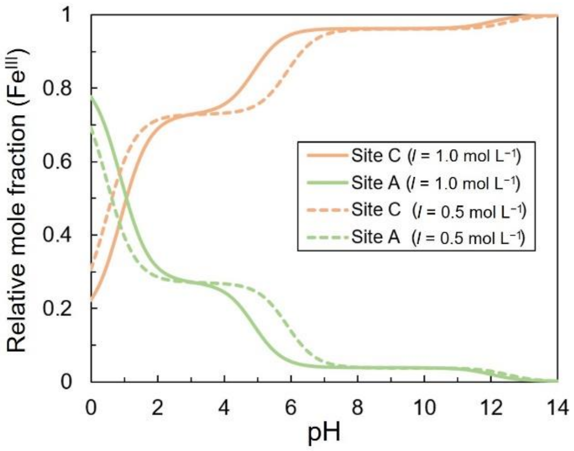

Figure S3. Comparison of the predicted QUE site selectivity for Fe^{III} as a function of pH using reference hydrolysis data from Ref [15] taken at I = 1.0 (solid lines) and 0.5 mol L^{-1} (dashed lines).

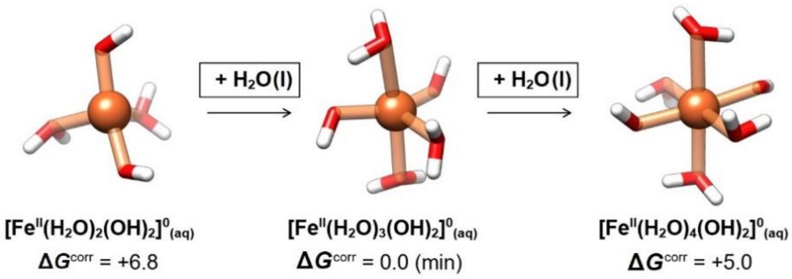

Figure S4. Optimized structures and relative Gibbs free energies (in kJ mol^{-1}) for the $[Fe^{II}(H_2O)_2(OH)_2]^0$, $[Fe^{II}(H_2O)_3(OH)_2]^0$, and $[Fe^{II}(H_2O)_4(OH)_2]^0$ complexes.

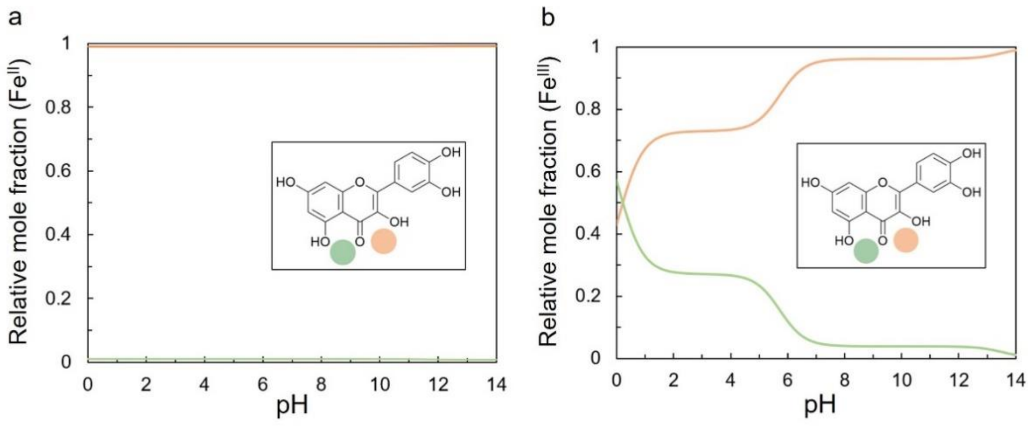

Figure S5. Comparison of the predicted QUE site selectivity for (a) Fe^{II} and (b) Fe^{III} as a function of pH.

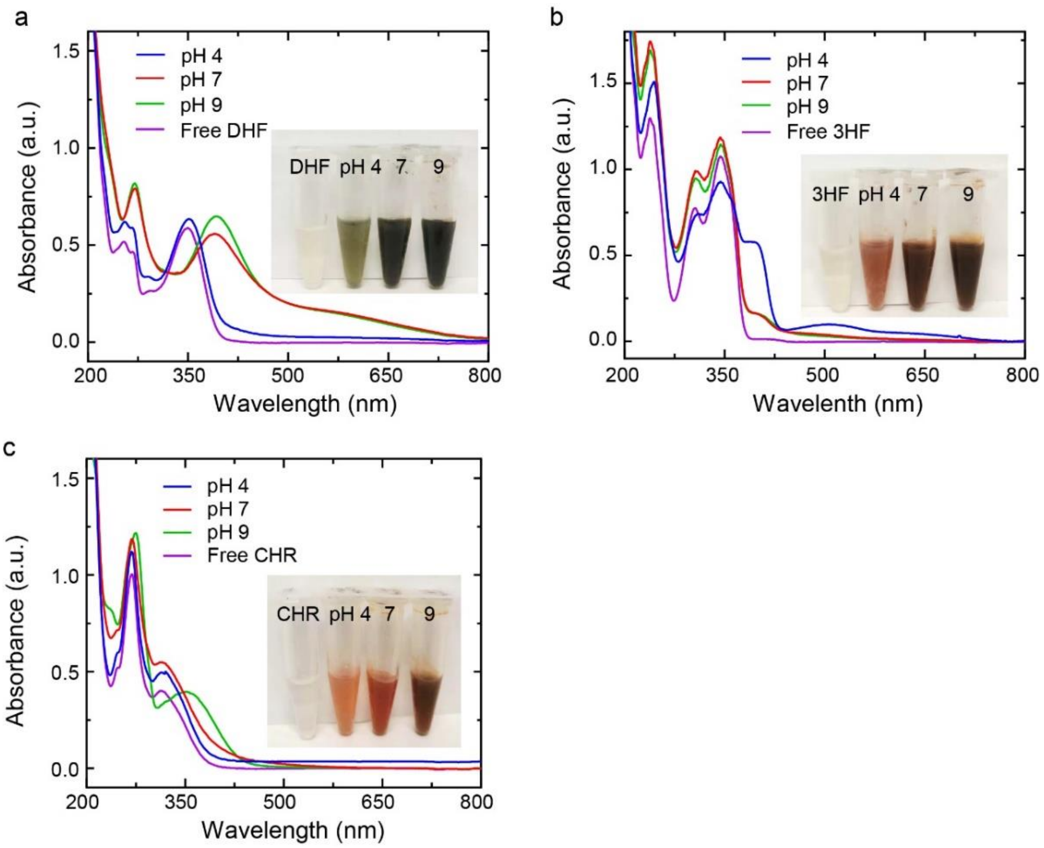

Figure S6. UV–vis spectra of (a) free DHF (featuring catechol group only) in methanol and Fe^{II}–DHF complexes at different pH, (b) free 3HF (featuring maltol group only) in methanol and Fe^{II}–3HF at different pH, and (c) free CHR (featuring acetylacetone group only) in methanol and Fe^{II}–CHR complexes at different pH. A bathochromic shift was observed in Fe^{II}–DHF complexes at pH 7 and pH 9. The absorption of Fe^{II}–3HF complexes at pH 4, 7, and 9 was shifted. The bathochromic shift in Fe^{II}–CHR complexes was observed at pH 9.

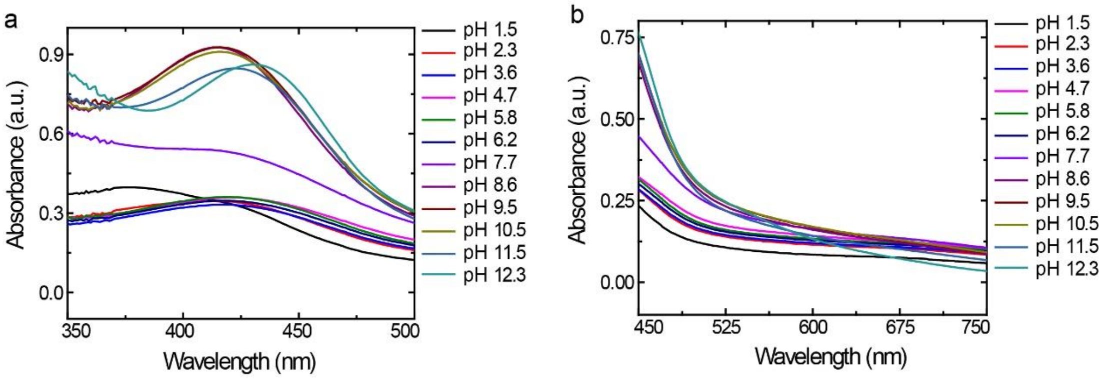

Figure S7. UV–vis spectra of (a) Band I and (b) Band II in Fe^{II}–QUE complexes as a function of pH.

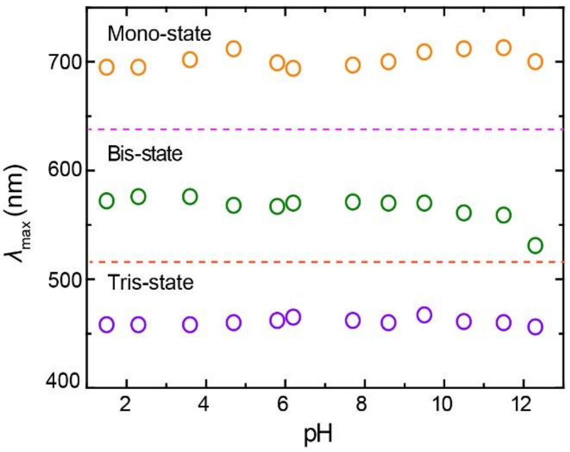

Figure S8. Maximum absorption wavelength (λ_{max}) of Fe^{II}–QUE complexes as a function of pH. Mono-state was identified at wavelength 640–720 nm, bis-state was identified at wavelength 520–640 nm, and tris-state was identified at wavelength 400–520 nm.

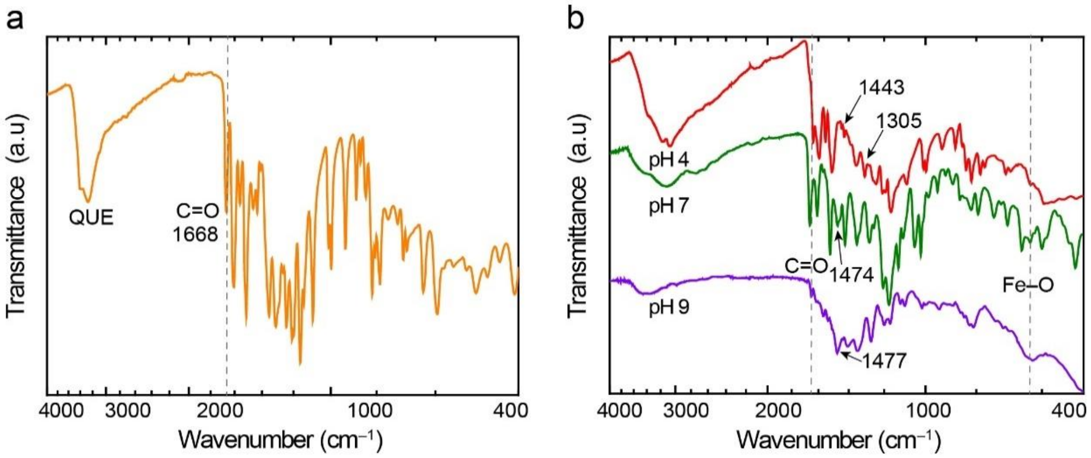

Figure S9. FTIR spectra of (a) free QUE and (b) Fe^{II}–QUE complexes at pH 4, 7, and 9. The spectra signal of the complexes at 625 cm^{-1} was ascribed to Fe–O stretching. The aryl ketonic stretching C=O at 1668 cm^{-1} for quercetin shifted to 1647 cm^{-1} for Fe^{II}–QUE complexes, implying coordination between the carbonyl oxygen and metal ions. The vibrations at 1443 and 1305 cm^{-1} were assigned to ring deformation with strong contribution of C–H and O–H bending. The signals at 1474 and 1477cm^{-1} were attributed to the C–C stretching in the phenolic groups.

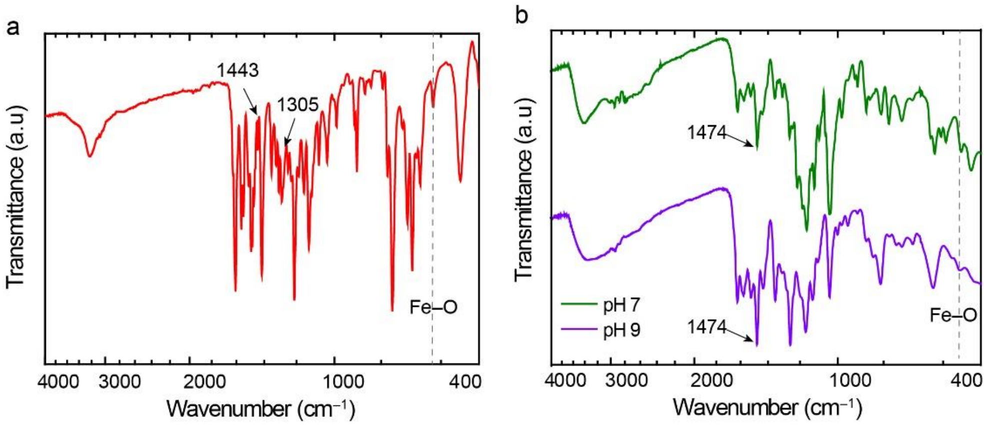

Figure S10. (a) FTIR spectrum of Fe^{II}–3HF complexes at pH 4. The vibrations at 1443 and 1305 cm^{-1} were assigned to ring deformation with a strong contribution of C–H and O–H bending. (b) FTIR spectrum of Fe^{II}–DHF complexes at pH 7 and 9. The signal at 1474 cm^{-1} was attributed to C–C stretching in the phenolic groups.

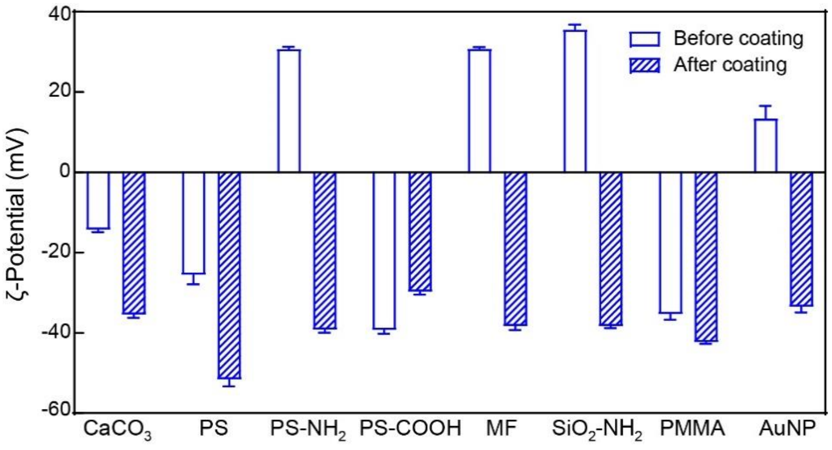

Figure S11. ζ-Potential values of particle substrates measured in water before and after MPN coating.

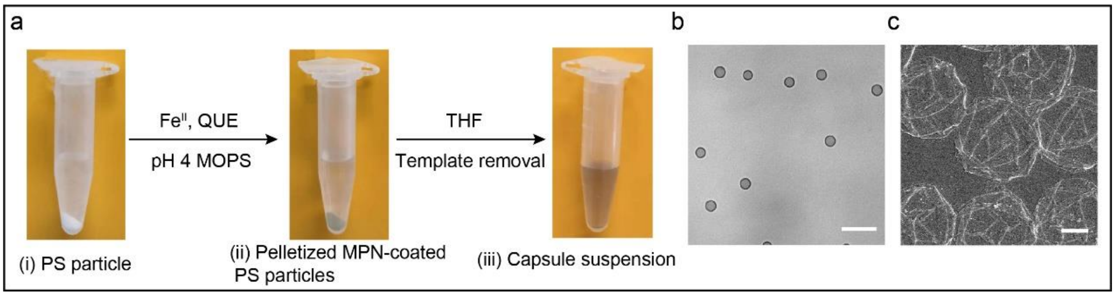

Figure S12. (a) Synthesis of MPN capsules at pH 4 MOPS. Microscopy images showing (b) the well-dispersed capsules in water and (c) the air-dried capsules. Scale bars are 10 µm (b) and 2 µm (c).

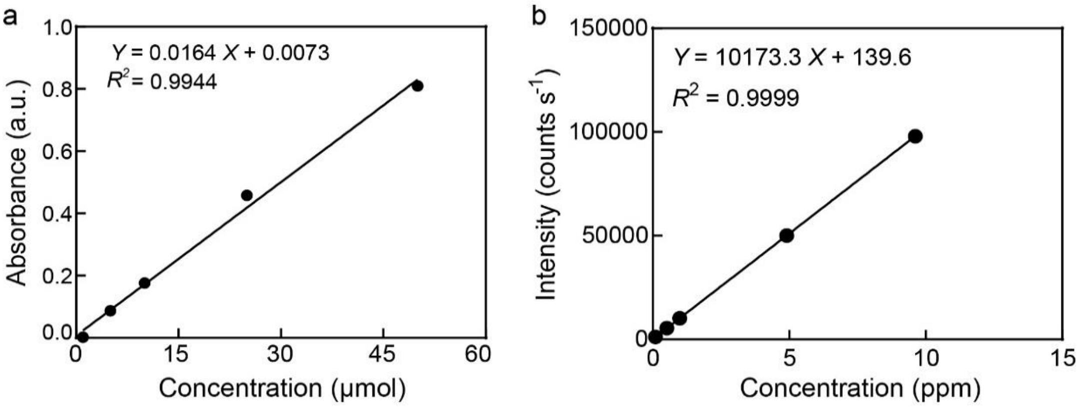

Figure S13. (a) UV–vis standard curve of QUE concentration at 371 nm. (b) ICP-OES standard curve of Fe intensity at a wavelength of 234 nm.

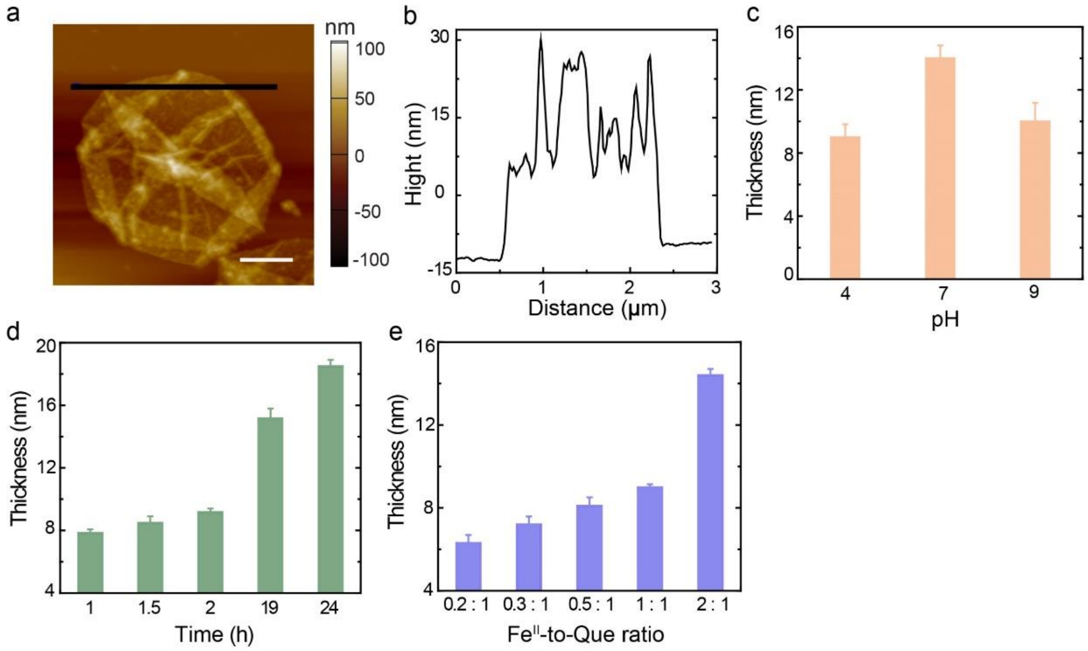

Figure S14. (a) Representative AFM image and (b) corresponding height profile of MPN_{QUE} capsules prepared at pH 4. Film thickness of MPN_{QUE} capsules obtained under different (c) pH, (d) incubation time, and (e) metal-to-ligand (Fe^{II}-to-QUE) ratio.

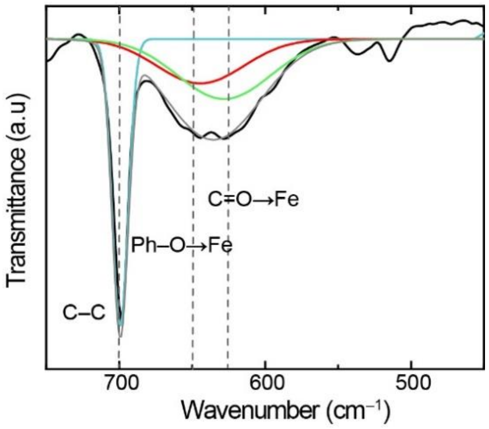

Figure S15. Peak fitting method applied for MPN_{QUE} capsules prepared at pH 4. The Fe–O binding to the carbonyl (C=O→Fe) was determined at 628 nm; the Fe–O binding to the phenyl (Ph–O→Fe) was determined at 645 nm; and the C–H stretching in the phenolic groups was determined at 700 nm.

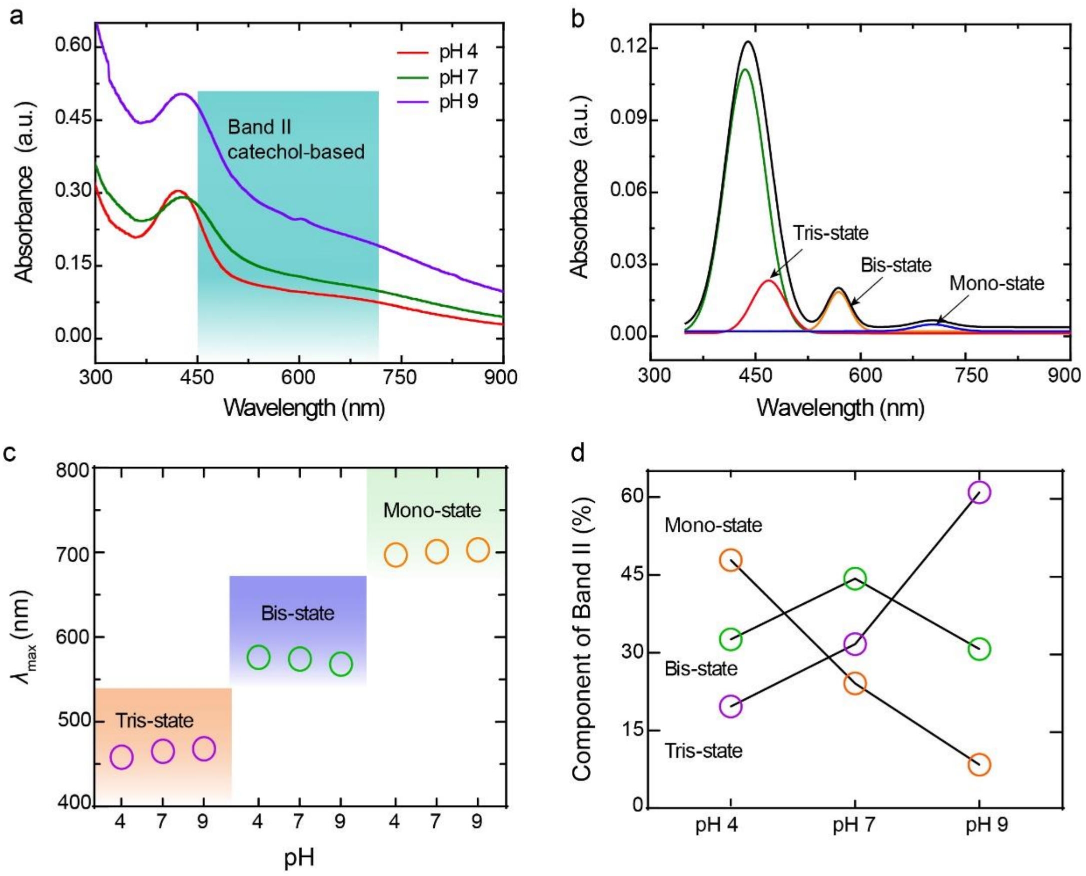

Figure S16. (a) UV–vis spectra of the catechol-based Band II of MPN_{QUE} capsules. (b) Peak fitting of MPN_{QUE} capsules prepared at pH 9. The black line represents the cumulative curve. The three identified peaks indicate the dominant mono-, bis-, and tris-state. (c) Maximum absorption of MPN_{QUE} capsules determined in the wavelength range of 400–800 nm. (d) Percentage of mono-, bis-, and tris-state determined for Band II of MPN_{QUE} capsules.

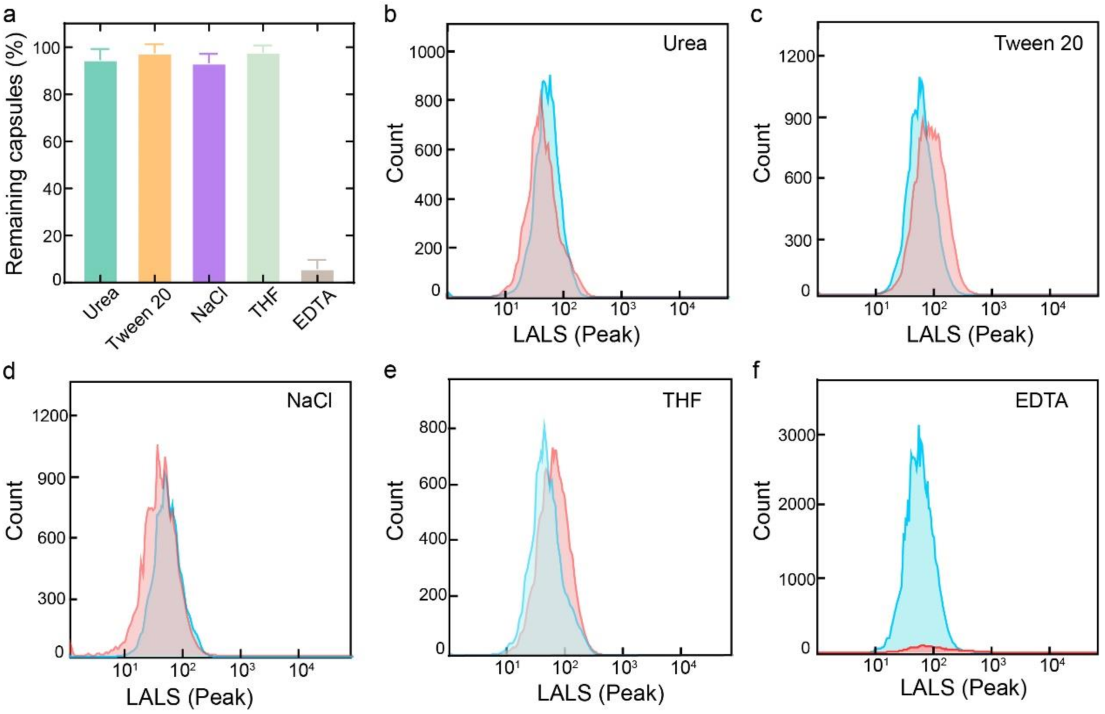

Figure S17. (a) Possible driving forces for MPN_{QUE} capsule assembly. Histograms of the disassembly of MPN_{QUE} capsules in (b) 100 mM Urea, (c) 100 mM Tween 20, (d) 100 mM NaCl, (e) THF, and (f) 100 mM EDTA, as determined by flow cytometry. The blue curve was measured at 0 h and the red curve was measured at 1.5 h. LALS means low-angle light scattering, representing the size of capsules.

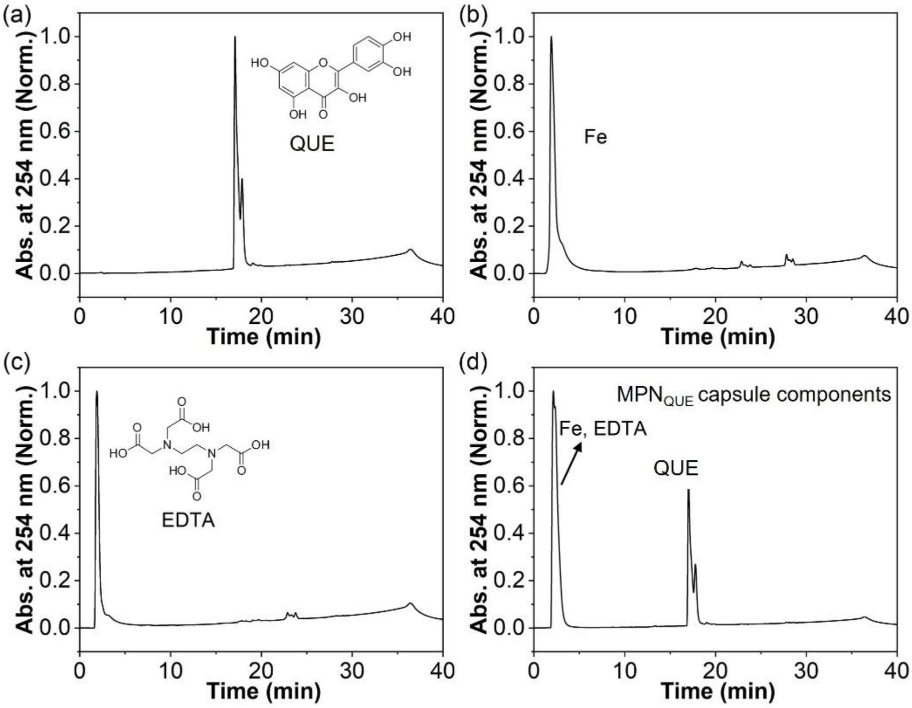

Figure S18. HPLC chromatograms of (a) QUE (retention time (RT) = 17.1 min), (b) Fe ion (RT = 1.9 min), (c) EDTA (RT = 1.9 min), and (d) MPN_{QUE} capsule components after degradation of the MPN_{QUE} capsules by EDTA.

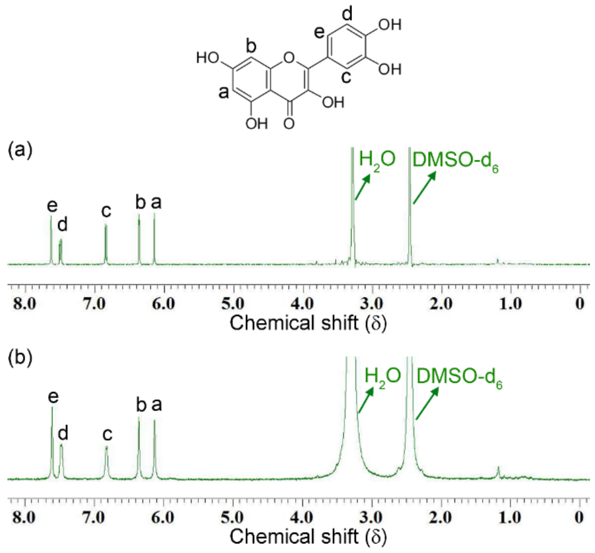

Figure S19. ^{1}H NMR spectra of (a) QUE and (b) MPN_{QUE} capsule components after degradation of the MPN_{QUE} capsules by EDTA.

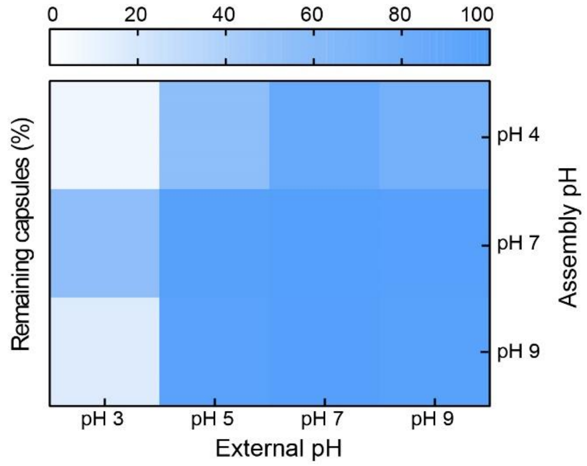

Figure S20. Heat map showing the disassembly kinetics of MPN_{QUE} capsules at different pH, including 100 mM glycine–HCl (pH 3), 100 mM sodium acetate (pH 5), 100 mM MOPS (pH 7), and 100 mM MOPS (pH 9). The capsules remaining after the disassembly experiments were assessed by flow cytometry, and the incubation time was 180 min.

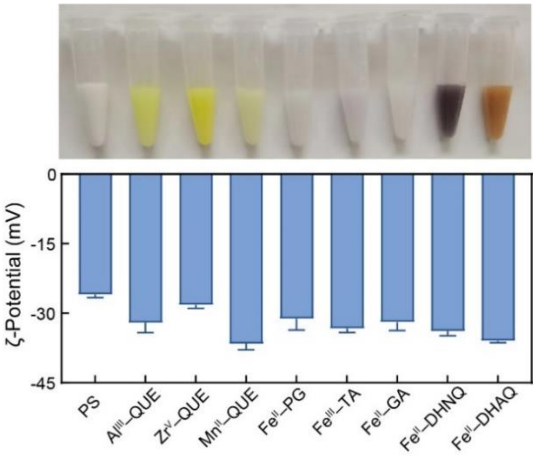

Figure S21. Photograph and ζ-potential values of PS and PS@MPN fabricated from different ligands and metal ions.

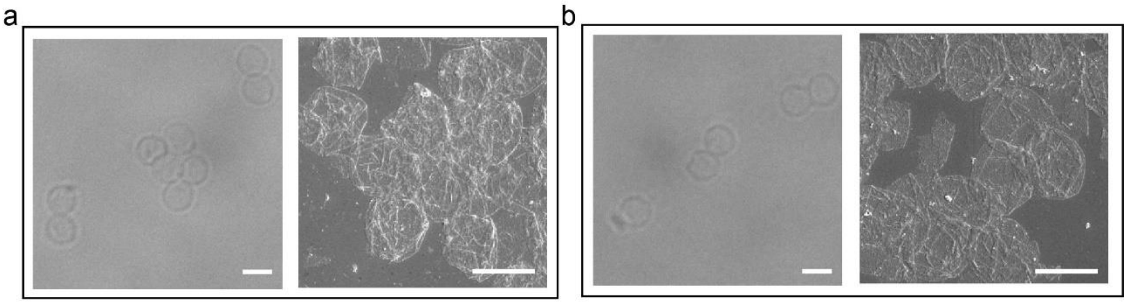

Figure S22. DIC and SEM images of (a) Zr^{IV}–QUE and (b) Al^{III}–QUE MPN capsules prepared at pH 4. Scale bars are 3 µm.

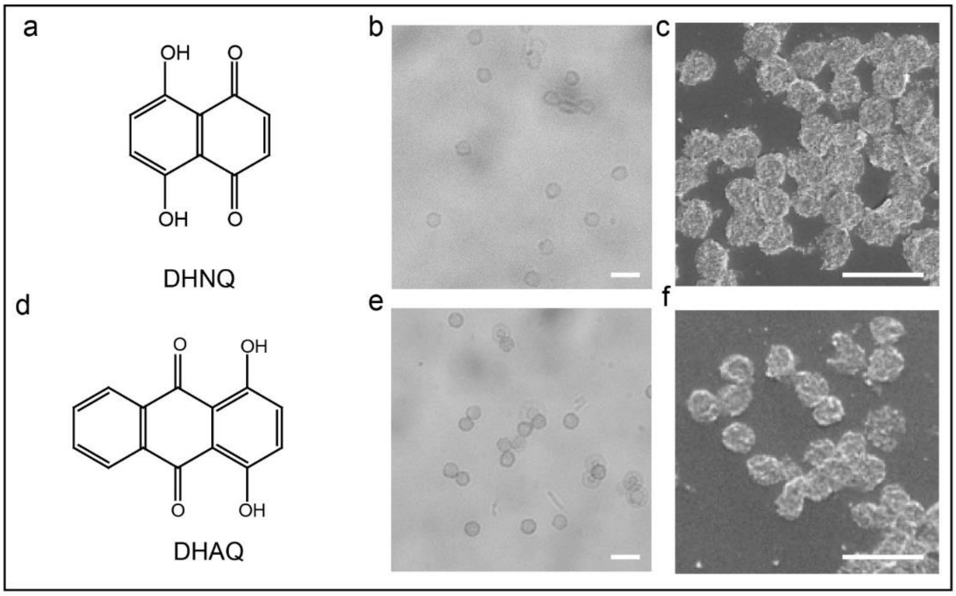

Figure S23. Chemical structures of (a) DHNQ and (d) DHAQ. (b) DIC and (c) SEM images of Fe^{II}–DHNQ capsules. (e) DIC and (f) SEM images of Fe^{II}–DHAQ capsules. Scale bars are 5 µm.

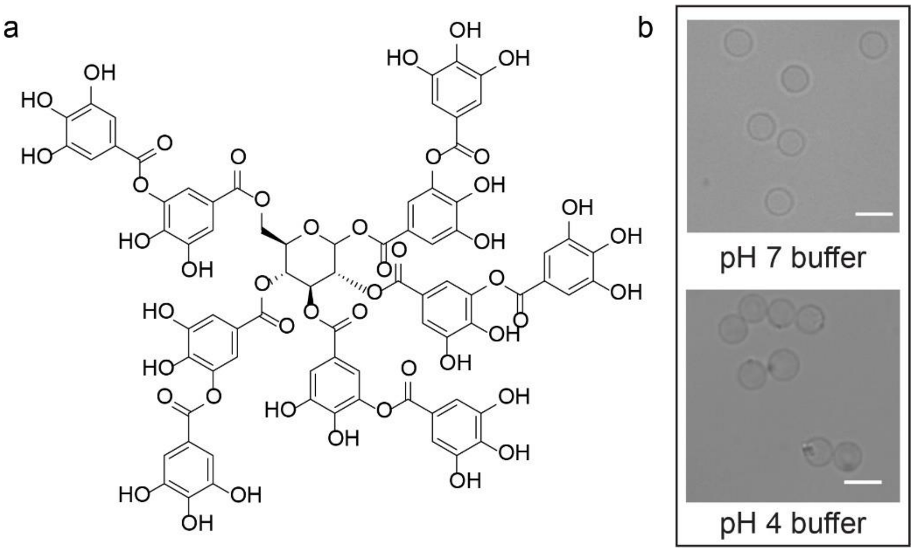

Figure S24. (a) Chemical structure of TA. (b) DIC images of Fe^{II}–TA MPN capsules dispersed in pH 7 (left) and pH 4 (right) buffer. Scale bars are 5 µm.

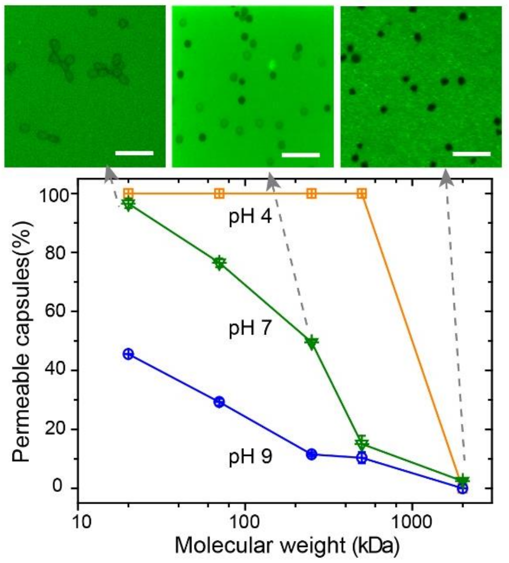

Figure S25. Representative CLSM images and percentage of permeable MPN_{QUE} capsules assembled at different pH as a function of the M_w of FITC–dextran.

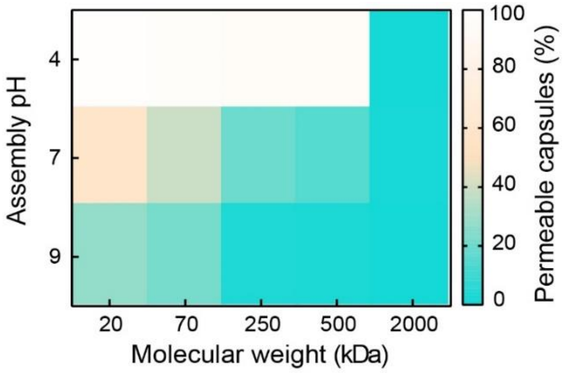

Figure S26. Permeability of Fe^{III}–QUE MPN capsules assembled at different pH toward FITC–dextran of varying M_w of 20–2000 kDa.

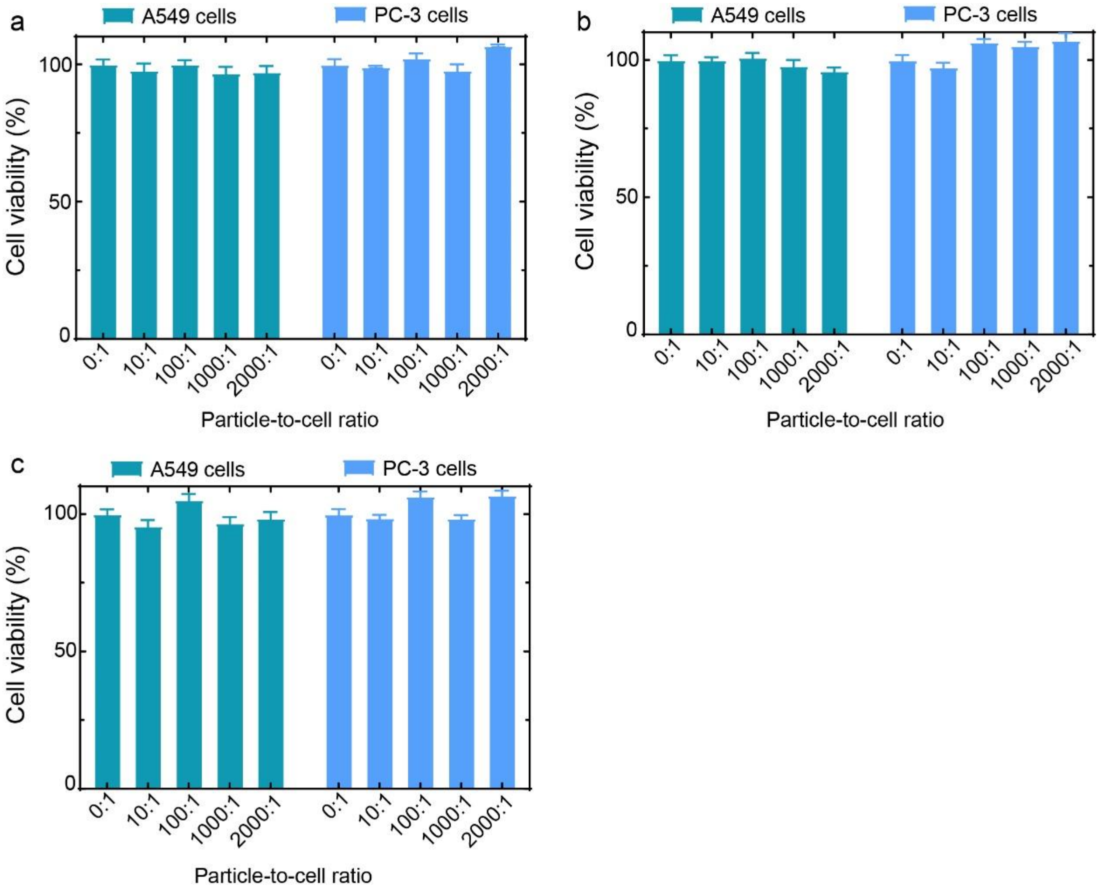

Figure S27. Cytotoxicity of MPN_{QUE} capsules prepared at (a) pH 4, (b) pH 7, and (c) pH 9 as a function of the particle-to-cell ratio. Cell viability was evaluated by XTT assay (mean ± SD, *n* = 3).

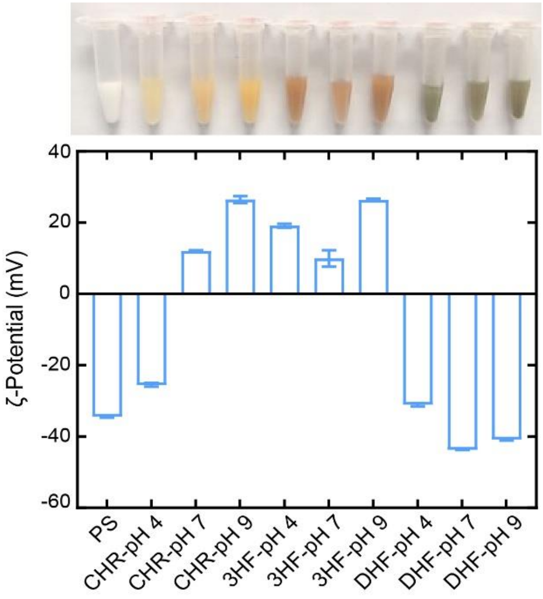

Figure S28. ζ-Potential values of PS and different MPN coatings measured in water. A photograph of the suspensions of the different coatings is also shown. The coatings formed are denoted as “flavonoid-pH”. For instance, CHR-pH 4 represents the coating obtained from coordination of Fe^{II} and CHR at pH 4.

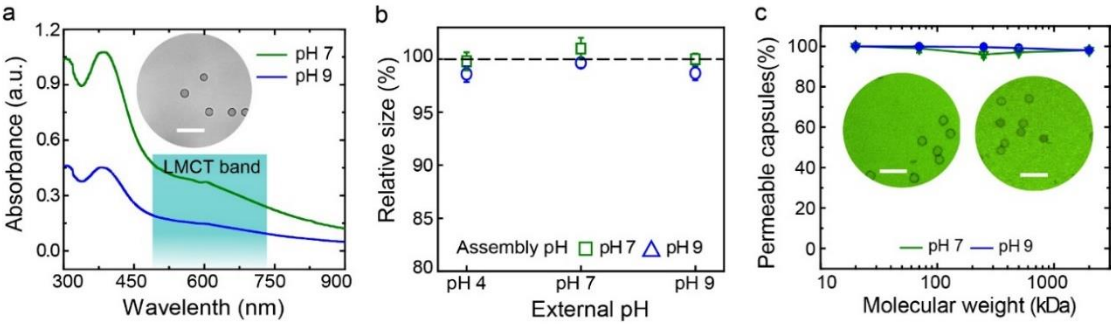

Figure S29. (a) UV–vis spectra of MPN_{DHF} capsules (obtained upon coordination of Fe^{II} and DHF at pH 7 or 9). The presence of the ligand-to-metal charge transfer (LMCT) band suggests the occurrence of metal coordination during capsule formation. Inset shows a representative DIC image of the capsules. (b) Relative size of MPN_{DHF} capsules at pH 7 or pH 9 MOPS (100 mM). The dotted line represents the template size (i.e., 3.2 μm normalized to 100%) and the percentage size of the capsules is measured relative to the template size (100%). (c) Percentage of MPN_{DHF} capsules permeable toward FITC–dextran of varying M_w of 20–2000 kDa. Capsules were prepared at pH 7 (green line) or pH 9 (blue line). Scale bars are 10 μm.

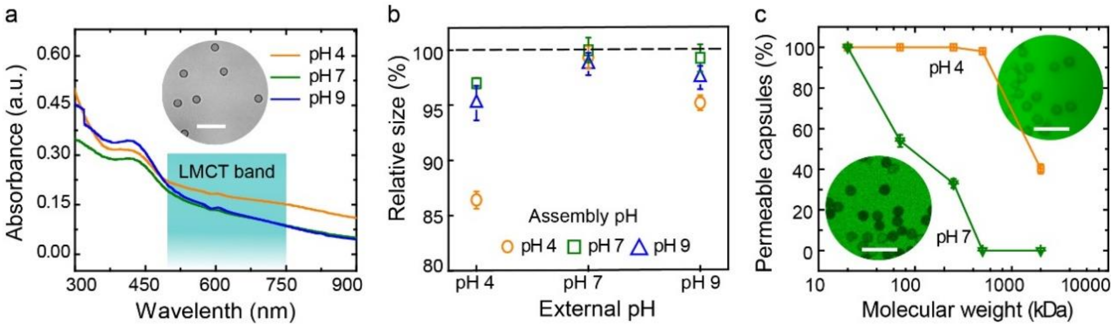

Figure S30. (a) UV–vis spectra of MPN_{FIS} capsules (prepared upon coordination of Fe^{II} and FIS at pH 4, 7, or 9). The presence of the LMCT band suggests the occurrence of metal coordination during capsule formation. Inset shows a representative DIC image of the capsules. (b) Relative size of MPN_{FIS} capsules at pH 4, pH 7, or pH 9 MOPS (100 mM). The dotted line represents the template size (i.e., 3.2 μm normalized at 100%), and the percentage size of the capsules is measured relative to the template size (100%). (c) Percentage of MPN_{FIS} capsules permeable toward FITC–dextran of varying M_w of 20–2000 kDa. The capsules were prepared at pH 4 (orange line) or pH 7 (green line). Scale bars are 10 μm.

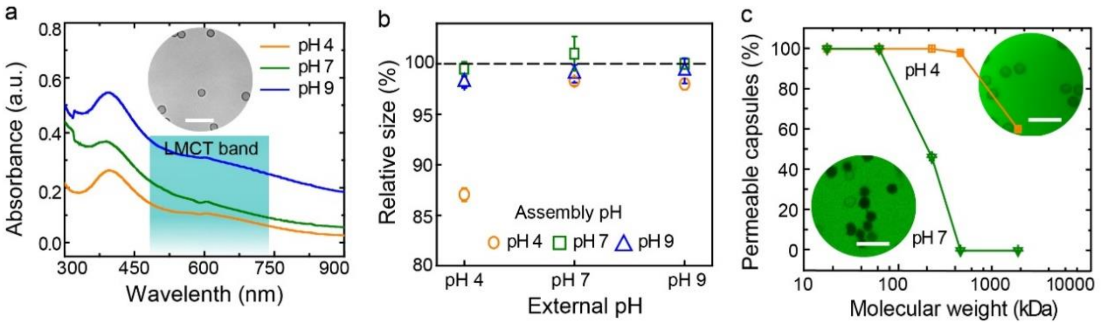

Figure S31. (a) UV–vis spectra of MPN_{LUT} capsules (obtained upon coordination of Fe^{II} and LUT at pH 4, 7, or 9). The presence of the LMCT band suggests the occurrence of metal coordination during capsule formation. Inset shows a representative DIC image of the capsules. (b) Relative size of MPN_{LUT} capsules at pH 4, pH 7, or pH 9 MOPS (100 mM). The dotted line represents the template size (i.e., 3.2 μm normalized at 100%), and the percentage size of the capsules is measured relative to the template size (100%). (c) Percentage of MPN_{LUT} capsules permeable toward FITC–dextran of varying M_w of 20–2000 kDa. Capsules were prepared at pH 4 (orange line) or pH 7 (green line). Scale bars are 10 μm.

Section S3. Supplementary Tables

Table S1. Gibbs free energies for binding of $[Fe^{II}(H_2O)_6]^{2+}$ to QUE (Site A) or CHR, and pK_a values of the resultant complexes[a]

Species	$\Delta G_{B/LH}$ (kJ mol^{-1})	ΔG_B* (kJ mol^{-1})	$\Delta G_{A/MLH}$ (kJ mol^{-1})	$-\log_{10}(K_B*)$	pK_a
$[Fe^{II}(H_2O)_4(QUE)]^{2+}$	38.2	-	−25.3	-	−4.4
$[Fe^{II}(H_2O)_4$(QUE-anion)$]^{+}$	-	12.9	66.8	2.3	11.7
$[Fe^{II}(H_2O)_2$(OH)(QUE-anion)$]^{0}$	-	79.7	-	14.0	-
$[Fe^{II}(H_2O)_4(CHR)]^{2+}$	34.9	-	−21.3	-	−3.7
$[Fe^{II}(H_2O)_4$(CHR-anion)$]^{+}$	-	13.5	70.7	2.4	12.4
$[Fe^{II}(H_2O)_2$(OH)(CHR-anion)$]^{0}$	-	84.2	-	14.7	-

[a] Calculated at 25 °C.

Table S2. Gibbs free energies for binding of $[Fe^{II}(H_2O)_6]^{2+}$ to QUE (Site C) or 3HF, and pK_a values of the resultant complexes[a]

Species	$\Delta G_{B/LH}$ (kJ mol^{-1})	ΔG_B* (kJ mol^{-1})	$\Delta G_{A/MLH}$ (kJ mol^{-1})	$-\log_{10}(K_B*)$	pK_a
$[Fe^{II}(H_2O)_4(QUE)]^{2+}$	31.8	-	−30.4	-	−5.3
$[Fe^{II}(H_2O)_4$(QUE-anion)$]^{+}$	-	1.4	66.3	0.2	11.6
$[Fe^{II}(H_2O)_2$(OH)(QUE-anion)$]^{0}$	-	67.7	-	11.9	-
$[Fe^{II}(H_2O)_4(3HF)]^{2+}$	26.5	-	−28.7	-	−5.0
$[Fe^{II}(H_2O)_4$(3HF-anion)$]^{+}$	-	−2.2	69.8	−0.4	12.2
$[Fe^{II}(H_2O)_2$(OH)(3HF-anion)$]^{0}$	-	67.6	-	11.8	-

[a] Calculated at 25 °C.

Table S3. Gibbs free energies for binding of $[Fe^{II}(H_2O)_6]^{2+}$ to QUE (Site B) or DHF, and pK_a values of the resultant complexes[a]

Species	$\Delta G_{B/LH}$ (kJ mol^{-1})	ΔG_B* (kJ mol^{-1})	$\Delta G_{A/MLH}$ (kJ mol^{-1})	$-\log_{10}(K_B*)$	pK_a
$[Fe^{II}(H_2O)_4(QUE)]^{2+}$	52.6	-	−25.7	-	−4.5
$[Fe^{II}(H_2O)_4$(QUE-anion)$]^{+}$	-	26.9	45.3	4.7	7.9
$[Fe^{II}(H_2O)_3$(QUE-dianion)$]^{0}$	-	72.2	-	12.6	-
$[Fe^{II}(H_2O)_4(DHF)]^{2+}$	55.2	-	−29.0	-	−5.1
$[Fe^{II}(H_2O)_4$(DHF-anion)$]^{+}$	-	26.2	43.1	4.6	7.6
$[Fe^{II}(H_2O)_3$(DHF-dianion)$]^{0}$	-	69.3	-	12.1	-

[a] Calculated at 25 °C.

Table S4. Effective Gibbs free energies for binding and spontaneous deprotonation of the resultant metal–ligand complexes with respect to $[Fe^{II}(H_2O)_6]^{2+}$ and the dominant metal–ligand complex at the indicated pH[a]

**Ligand (Site)**	**Effective ΔG_B*(kJ mol^{-1}) relative to $[Fe^{II}(H_2O)_6]^{2+}$ complex**		
	**pH 4**	**pH 7**	**pH 9**
QUE (A)	−9.9	−27.0	−38.4
CHR	−9.3	−26.4	−37.8
QUE (C)	−21.4	−38.6	−50.0
3HF	−25.0	−42.1	−53.5
QUE (B)	4.0	−13.3	−30.8
DHF	3.3	−14.4	−33.5

Color scale:

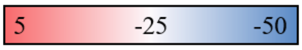

[a] Estimated by assuming a Boltzmann population of the respective protonation states of the complexes at each pH value and calculated at 25 °C.

Table S5. Gibbs free energies for binding of $[Fe^{III}(H_2O)_6]^{3+}$ to QUE (Site A) or CHR, and pK_a values of the resultant complexes[a]

**Species**	**$\Delta G_{B/LH}$ (kJ mol^{-1})**	**ΔG_B* (kJ mol^{-1})**	**$\Delta G_{A/MLH}$ (kJ mol^{-1})**	**$-\log_{10}(K_B$*)**	**pK_a**
$[Fe^{III}(H_2O)_4(QUE)]^{3+}$	56.7	-	−59.4	-	−10.4
$[Fe^{III}(H_2O)_4$(QUE-anion)$]^{2+}$	-	−2.7	3.1	-0.5	0.6
$[Fe^{III}(H_2O)_3(OH)$(QUE-anion)$]^{+}$	-	0.3	37.5	0.1	6.6
$[Fe^{III}(H_2O)(OH)_2$(QUE-anion)$]^{0}$	-	37.8	85.2	6.6	14.9
$[Fe^{III}(OH)_3$(QUE-anion)$]^{-}$	-	123.1	-	21.6	-
$[Fe^{III}(H_2O)_4(CHR)]^{3+}$	28.1	-	−44.5	-	−7.8
$[Fe^{III}(H_2O)_4$(CHR-anion)$]^{2+}$	-	−16.4	16.6	−2.9	2.9
$[Fe^{III}(H_2O)_3(OH)$(CHR-anion)$]^{+}$	-	0.2	35.6	0.0	6.2
$[Fe^{III}(H_2O)(OH)_2$(CHR-anion)$]^{0}$	-	35.8	91.1	6.3	16.0
$[Fe^{III}(OH)_3$(CHR-anion)$]^{-}$	-	126.9	-	22.2	-

[a] Calculated at 25 °C.

Table S6. Gibbs free energies for binding of $[Fe^{III}(H_2O)_6]^{3+}$ to QUE (Site C) or 3HF, and pK_a values of the resultant complexes[a]

Species	$\Delta G_{B/LH}$ (kJ mol^{-1})	ΔG_B* (kJ mol^{-1})	$\Delta G_{A/MLH}$ (kJ mol^{-1})	$-\log_{10}(K_B$*)	pK_a
$[Fe^{III}(H_2O)_4(QUE)]^{3+}$	68.5	-	−67.1	-	−11.8
$[Fe^{III}(H_2O)_4$(QUE-anion)$]^{2+}$	-	1.4	−3.5	0.2	−0.6
$[Fe^{III}(H_2O)_3(OH)$(QUE-anion)$]^{+}$	-	−2.1	32.0	−0.4	5.6
$[Fe^{III}(H_2O)(OH)_2$(QUE-anion)$]^{0}$	-	29.8		5.2	13.5
$[Fe^{III}(OH)_3$(QUE-anion)$]^{-}$	-	106.9	-	18.7	-
$[Fe^{III}(H_2O)_4(3HF)]^{3+}$	34.3	-	−49.0	-	−8.6
$[Fe^{III}(H_2O)_4$(3HF-anion)$]^{2+}$	-	−14.7	5.6	−2.6	1.0
$[Fe^{III}(H_2O)_3(OH)$(3HF-anion)$]^{+}$	-	−9.1	34.1	−1.6	6.0
$[Fe^{III}(H_2O)(OH)_2$(3HF-anion)$]^{0}$	-	25.0	81.6	4.4	14.3
$[Fe^{III}(OH)_3$(3HF-anion)$]^{-}$	-	106.7	-	18.7	-

[a] Calculated at 25 °C.

Table S7. Gibbs free energies) for binding of $[Fe^{III}(H_2O)_6]^{3+}$ to QUE (Site B) or DHF, and pK_a values of the resultant complexes[a]

Species	$\Delta G_{B/LH}$ (kJ mol^{-1})	ΔG_B* (kJ mol^{-1})	$\Delta G_{A/MLH}$ (kJ mol^{-1})	$-\log_{10}(K_B$*)	pK_a
$[Fe^{III}(H_2O)_4(QUE)]^{3+}$	141.1	-	−84.1	-	−14.7
$[Fe^{III}(H_2O)_4$(QUE-anion)$]^{2+}$	-	57.0	−31.3	10.0	−5.5
$[Fe^{III}(H_2O)_4$(QUE-dianion)$]^{+}$	-	25.7	15.7	4.5	2.8
$[Fe^{III}(H_2O)_2(OH)$(QUE-dianion)$]^{0}$	-	41.4	47.9	7.3	8.4
$[Fe^{III}(H_2O)(OH)_2$(QUE-anion)$]^{-}$	-	89.4	-	15.7	-
$[Fe^{III}(H_2O)_4(DHF)]^{3+}$	115.9	-	−60.6	-	−10.6
$[Fe^{III}(H_2O)_4$(DHF-anion)$]^{2+}$	-	55.4	−21.3	9.7	−3.7
$[Fe^{III}(H_2O)_4$(DHF-dianion)$]^{+}$	-	34.1	3.2	6.0	0.6
$[Fe^{III}(H_2O)_2(OH)$(DHF-dianion)$]^{0}$	-	37.2	51.6	6.5	9.0
$[Fe^{III}(H_2O)(OH)_2$(DHF-anion)$]^{-}$	-	88.8	-	15.6	-

[a] Calculated at 25 °C.

Table S8. Effective Gibbs free energies for binding and spontaneous deprotonation of the resultant metal–ligand complexes with respect to $[Fe^{III}(H_2O)_6]^{3+}$ and the dominant metal–ligand complex at the indicated pH[a]

Ligand (Site)	Effective ΔG_B* (kJ mol^{-1}) relative to the $[Fe^{III}(H_2O)_6]^{3+}$ complex		
	pH 4	pH 7	pH 9
QUE (A)	−45.3	−82.8	−116.3
CHR	−45.5	−84.5	−118.3
QUE (C)	−47.9	−90.1	−124.3
3HF	−54.8	−95.1	−129.1
QUE (B)	−27.2	−78.5	−116.7
DHF	−31.3	−82.7	−119.5

Color scale:

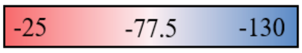

[a] Estimated by assuming a Boltzmann population of the respective protonation states of the complexes at each pH value and calculated at 25 °C.

Table S9. Relative Gibbs free energies for binding to Site A vs Site C of QUE[a]

Species	Site A (kJ mol^{-1})	Site C (kJ mol^{-1})
$[Fe^{III}(H_2O)_4(QUE)]^{3+}$	0.0 (min)	+11.9
$[Fe^{III}(H_2O)_4(QUE\text{-}anion)]^{3+}$	0.0 (min)	+4.1
$[Fe^{III}(H_2O)_3(OH)(QUE\text{-}anion)]^{3+}$	+2.5	0.0 (min)
$[Fe^{III}(H_2O)(OH)_2(QUE\text{-}anion)]^{3+}$	+8.0	0.0 (min)
$[Fe^{III}(OH)_3(QUE\text{-}anion)]^{3+}$	+14.7	0.0 (min)
$[Fe^{II}(H_2O)_4(QUE)]^{2+}$	+6.4	0.0 (min)
$[Fe^{II}(H_2O)_4(QUE\text{-}anion)]^{+}$	+11.5	0.0 (min)
$[Fe^{II}(H_2O)_2(OH)(QUE\text{-}anion)]^{0}$	+12.0	0.0 (min)

[a] Calculated at 25 °C.

Table S10. Integrated area of fitted peaks of Fe^{II}–QUE complexes at different pH[a]

pH	Int. area (Tris-state)	Int. area (Bis-state)	Int. area (Mono-state)
1.5	0.1	0.3	0.9
2.3	0.2	0.5	1.4
3.6	0.9	0.6	1.6
4.7	1.4	0.6	1.6
5.8	1.5	0.6	1.4
6.2	1.9	0.7	1.6
7.7	2.6	0.9	1.8
8.6	2.5	1	1.9
9.5	3.5	1.2	2.4
10.5	5.2	2	4.1
11.5	9.8	2.3	3.4
12.3	10.9	2.1	1.5

[a] Gaussian peak function was used in the fitting model.

Table S11. FTIR information of the detected characteristic bands of QUE, and Fe^{II}–QUE complexes prepared at pH 4, 7, and 9

Group assigned to given band	Wavenumber (cm^{-1})			
	QUE	pH 4	pH 7	pH 9
O–H stretching vibration of phenol	3401, 3277	3389, 3165	3417, 3100	3352, 3118
C=O aryl ketonic stretch	1668	1645	1647	1647
C=C aromatic ring stretching bands	1605, 1559, 1512, 1457, 1436	1593, 1551, 1507, 1443, 1428	1593, 1557, 1507, 1474, 1411	1566, 1474, 1411
O–H bending of phenol	1351	1356	1342	1349
C–H bonds in aromatic hydrocarbon bending (in-plane)	1309, 1094, 994, 932	1305, 1087, 1010, 997, 929	1116, 1092, 1013, 940	1115, 1095, 1018, 939
C–O stretching of aryl ether (C–O–C)	1241	1242	1262, 1244	1271
C–O stretching in phenol	1210	1207	1214	1201
C–CO–C stretching and bending in ketones	1161	1165	1165	1166
C–H bending of aromatic hydrocarbons (out-of-plane)	882, 842, 817, 786, 639	877, 851, 836, 785, 631, 595	906, 879, 852, 833, 781, 692, 648, 590	898, 855, 827, 737, 586
Fe–O stretching	-	625	624	625

Table S12. Concentrations of QUE and Fe^{II}, and the ligand-to-metal ratio of the MPN capsules

MPN capsules	Concentration of QUE (×10^{-9} μmol per capsule)	Concentration of Fe^{II} (×10^{-9} μmol per capsule)	Ratio of QUE to Fe^{II}
pH 4	14	5.9	2.3
pH 7	30	5	6.0
pH 9	34	8.9	3.8

Table S13. Percentage of integrated area of the Fe–O band[a]

MPN capsules	Int. area (%) (C=O→Fe)	Int. area (%) (Ph–O→Fe)
pH 4	59.2	40.8
pH 7	52.6	47.4
pH 9	50	50

[a] The Fe–O band of the MPN capsules can be attributed to Fe–O binding to the carbonyl (C=O→Fe) and Fe–O binding to the phenyl (Ph–O→Fe)

Table S14. Integrated area of fitted peaks of the MPN capsules prepared at different pH[a]

MPN capsules	Int. area (Tris-state)	Int. area (Bis-state)	Int. area (Mono-state)
pH 4	0.5	0.83	1.22
pH 7	0.25	0.35	0.19
pH 9	1.31	0.66	0.18

[a] The Gaussian peak function was used in the fitting model.

Raw energies of all the species in this study

Table S15. Free ligands (all units are in Hartree)

Species	E_{sol} B97MV/ def2-TZVP	G_{sol} B97MV/ def2-TZVP	E_{sol} DLPNO-CCSD(T)/ cc-pVTZ	G_{sol} Final Gibbs energy (Water)
H_2O	−76.443081	−76.439382	−76.341762	−76.338063
QUE	−1104.567241	−1104.379639	−1102.460912	−1102.273311
CHR	−878.850982	−878.672256	−877.064254	−876.885529
3HF	−803.595644	−803.419947	−801.915055	−801.739358
DHF	−878.840134	−878.662495	−877.053726	−876.876087

Table S16. Neutral and cationic Fe^{II} complexes (all units are in Hartree)

Species	E_{sol} B97MV/ def2-TZVP	G_{sol} B97MV/ def2-TZVP	E_{sol} DLPNO-CCSD(T)/ cc-pVTZ	G_{sol} Final Gibbs energy (Water)
$[Fe^{II}(H_2O)_6]^{2+}$	−1721.93695	−1721.83328	−1720.94445	−1720.840783
$[Fe^{II}(H_2O)_5(OH)]^{+}$	−1721.47485	−1721.38395	−1720.47323	−1720.382328
$[Fe^{II}(H_2O)_3(OH)_2]^{0}$	−1644.55034	−1644.49418	−1643.64315	−1643.586983
$[Fe^{II}(H_2O)_4(OH)_2]^{0}$	−1721.003917	−1720.924779	−1719.995480	−1719.916343
$[Fe^{II}(H_2O)_2(OH)_2]^{0}$	−1568.097183	−1568.061423	−1567.288892	−1567.253132
$[Fe^{II}(H_2O)_4(CHR)]^{2+}$	−2447.89389	−2447.63012	−2445.31129	−2445.047515
$[Fe^{II}(H_2O)_4(CHR\text{-anion})]^{+}$	−2447.45749	−2447.20673	−2444.86862	−2444.617861
$[Fe^{II}(H_2O)_2(OH)(CHR\text{-anion})]^{0}$	−2370.53210	−2370.31573	−2368.03611	−2367.819736
$[Fe^{II}(H_2O)_4(QUE_A)]^{2+}$	−2673.60963	−2673.33614	−2670.70752	−2670.434026
$[Fe^{II}(H_2O)_4(QUE_A\text{-anion})]^{+}$	−2673.17353	−2672.91341	−2670.26599	−2670.005864
$[Fe^{II}(H_2O)_2(OH)(QUE_A\text{-anion})]^{0}$	−2596.24880	−2596.02383	−2593.43417	−2593.209207
$[Fe^{II}(H_2O)_4(3HF)]^{2+}$	−2372.63839	−2372.37786	−2370.16505	−2369.904523
$[Fe^{II}(H_2O)_4(3HF\text{-anion})]^{+}$	−2372.20465	−2371.95768	−2369.72464	−2369.477666
$[Fe^{II}(H_2O)_2(OH)(3HF\text{-anion})]^{0}$	−2295.28057	−2295.06695	−2292.8935	−2292.67988
$[Fe^{II}(H_2O)_4(QUE_C)]^{2+}$	−2673.60959	−2673.33644	−2670.70961	−2670.436468
$[Fe^{II}(H_2O)_4(QUE_C\text{-anion})]^{+}$	−2673.174950	−2672.91542	−2670.26978	−2670.010259
$[Fe^{II}(H_2O)_2(OH)(QUE_C\text{-anion})]^{0}$	−2596.250900	−2596.02542	−2593.43927	−2593.213782
$[Fe^{II}(H_2O)_4(DHF)]^{2+}$	−2447.871170	−2447.60754	−2445.29394	−2445.030318
$[Fe^{II}(H_2O)_4(DHF\text{-anion})]^{+}$	−2447.439890	−2447.19014	−2444.85335	−2444.603598
$[Fe^{II}(H_2O)_3(DHF\text{-dianion})]^{0}$	−2370.52468	−2370.30921	−2368.03144	−2367.815966
$[Fe^{II}(H_2O)_4(QUE_B)]^{2+}$	−2673.59946	−2673.3252	−2670.70281	−2670.428546
$[Fe^{II}(H_2O)_4(QUE_B\text{-anion})]^{+}$	−2673.16668	−2672.90622	−2670.26102	−2670.000555
$[Fe^{II}(H_2O)_3(QUE_B\text{-dianion})]^{0}$	−2596.24974	−2596.02445	−2593.43737	−2593.212082

Table S17. Neutral and cationic Fe^{III} complexes (all units are in Hartree)

Species	E_{sol} B97MV/ def2-TZVP	G_{sol} B97MV/ def2-TZVP	E_{sol} DLPNO-CCSD(T)/ cc-pVTZ	G_{sol} Final Gibbs Energy (Water)
$[Fe^{III}(H_2O)_6]^{3+}$	−1721.716749	−1721.609725	−1720.717532	−1720.610509
$[Fe^{III}(H_2O)_5(OH)]^{2+}$	−1721.302537	−1721.205826	−1720.294916	−1720.198204
$[Fe^{III}(H_2O)_4(OH)_2]^{+}$	−1720.864420	−1720.780072	−1719.850456	−1719.766108
$[Fe^{III}(H_2O)_2(OH)_3]^{0}$	−1643.953014	−1643.904856	−1643.036373	−1642.988216
$[Fe^{III}(H_2O)_4(CHR)]^{3+}$	−2447.685933	−2447.417655	−2445.088096	−2444.819818
$[Fe^{III}(H_2O)_4(CHR\text{-anion})]^{2+}$	−2447.285925	−2447.032587	−2444.682540	−2444.429202
$[Fe^{III}(H_2O)_3(OH)(CHR\text{-anion})]^{+}$	−2446.841977	−2446.600186	−2444.240173	−2443.998382
$[Fe^{III}(H_2O)(OH)_2(CHR\text{-anion})]^{0}$	−2369.930437	−2369.724138	−2367.426974	−2367.220676
$[Fe^{III}(H_2O)_4(QUE_A)]^{3+}$	−2673.413494	−2673.137524	−2670.472692	−2670.196722
$[Fe^{III}(H_2O)_4(QUE_A\text{-anion})]^{2+}$	−2673.003507	−2672.740189	−2670.075099	−2669.811781
$[Fe^{III}(H_2O)_3(OH)(QUE_A\text{-anion})]^{+}$	−2672.557422	−2672.307487	−2669.636035	−2669.386100
$[Fe^{III}(H_2O)(OH)_2(QUE_A\text{-anion})]^{0}$	−2595.645880	−2595.430561	−2592.823003	−2592.607684
$[Fe^{III}(H_2O)_4(3HF)]^{3+}$	−2372.425333	−2372.159996	−2369.936620	−2369.671283
$[Fe^{III}(H_2O)_4(3HF\text{-anion})]^{2+}$	−2372.031507	−2371.779753	−2369.534130	−2369.282376
$[Fe^{III}(H_2O)_3(OH)(3HF\text{-anion})]^{+}$	−2371.590829	−2371.351919	−2369.094650	−2368.855740
$[Fe^{III}(H_2O)(OH)_2(3HF\text{-anion})]^{0}$	−2294.680041	−2294.476547	−2292.282109	−2292.078615
$[Fe^{III}(H_2O)_4(QUE_C)]^{3+}$	−2673.404773	−2673.127463	−2670.469514	−2670.192204
$[Fe^{III}(H_2O)_4(QUE_C\text{-anion})]^{2+}$	−2673.005724	−2672.742199	−2670.073729	−2669.810204
$[Fe^{III}(H_2O)_3(OH)(QUE_C\text{-anion})]^{+}$	−2672.562563	−2672.311051	−2669.638549	−2669.387038
$[Fe^{III}(H_2O)(OH)_2(QUE_C\text{-anion})]^{0}$	−2595.650309	−2595.434425	−2592.826610	−2592.610726
$[Fe^{III}(H_2O)_4(DHF)]^{3+}$	−2447.653504	−2447.388194	−2445.042235	−2444.776926
$[Fe^{III}(H_2O)_4(DHF\text{-anion})]^{2+}$	−2447.255080	−2447.001250	−2444.646257	−2444.392427
$[Fe^{III}(H_2O)_4(DHF\text{-dianion})]^{+}$	−2446.836433	−2446.596462	−2444.216003	−2443.976032
$[Fe^{III}(H_2O)_2(OH)(DHF\text{-dianion})]^{0}$	−2369.923232	−2369.717728	−2367.416201	−2367.210696
$[Fe^{III}(H_2O)_4(QUE_B)]^{3+}$	−2673.394366	−2673.119275	−2670.439655	−2670.164564
$[Fe^{III}(H_2O)_4(QUE_B\text{-anion})]^{2+}$	−2672.985198	−2672.721544	−2670.052668	−2669.789014
$[Fe^{III}(H_2O)_4(QUE_B\text{-dianion})]^{+}$	−2672.564371	−2672.312342	−2669.628462	−2669.376432
$[Fe^{III}(H_2O)_2(OH)(QUE_B\text{-dianion})]^{0}$	−2595.649789	−2595.433097	−2592.823000	−2592.606308

Section S4. Additional Information

Checklist

Minimum Information Reporting in Bio–Nano Experimental Literature

The MIRIBEL guidelines were introduced here: https://doi.org/10.1038/s41565-018-0246-4

The development of these guidelines was led by the ARC Centre of Excellence in Convergent Bio-Nano Science and Technology: https://www.cbns.org.au/. Any updates or revisions to this document will be made available here: http://doi.org/10.17605/OSF.IO/SMVTF. This document is made available under a CC-BY 4.0 license: https://creativecommons.org/licenses/by/4.0/.

The MIRIBEL guidelines were developed to facilitate reporting and dissemination of research in bio–nano science. Their development was inspired by various similar efforts:

- MIAME (microarray experiments): *Nat. Genet.* **29** (2001), 365; http://doi.org/10.1038/ng1201-365
- MIRIAM (biochemical models): *Nat. Biotechnol.* **23** (2005) 1509; http://doi.org/10.1038/nbt1156
- MIBBI (biology/biomedicine): *Nat. Biotechnol.* **26** (2008) 889; http://doi.org/10.1038/nbt.1411
- MIGS (genome sequencing): *Nat. Biotechnol.* **26** (2008) 541; http://doi.org/10.1038/nbt1360
- MIQE (quantitative PCR): *Clin. Chem.* **55** (2009) 611; http://doi.org/10.1373/clinchem.2008.112797
- ARRIVE (animal research): *PLOS Biol.* **8** (2010) e1000412; http://doi.org/10.1371/journal.pbio.1000412
- *Nature*'s reporting standards:
    - Life science: https://www.nature.com/authors/policies/reporting.pdf; e.g., *Nat. Nanotechnol.* **9** (2014) 949; http://doi.org/10.1038/nnano.2014.287
    - Solar cells: https://www.nature.com/authors/policies/solarchecklist.pdf; e.g., *Nat. Photonics* **9** (2015) 703; http://doi.org/10.1038/nphoton.2015.233
    - Lasers: https://www.nature.com/authors/policies/laserchecklist.pdf; e.g., *Nat. Photonics* **11** (2017) 139; http://doi.org/10.1038/nphoton.2017.28
- The "TOP guidelines": e.g., *Science* **352** (2016) 1147; http://doi.org/10.1126/science.aag2359

Similar to many of the efforts listed above, the parameters included in this checklist are **not** intended to be definitive requirements; instead they are intended as 'points to be considered', with authors themselves deciding which parameters are—and which are not—appropriate for their specific study.

This document is intended to be a living document, which we propose is revisited and amended annually by interested members of the community, who are encouraged to contact the authors of this document. Parts of this document were developed at the annual International Nanomedicine Conference in Sydney, Australia: http://www.oznanomed.org/, which will continue to act as a venue for their review and development, and interested members of the community are encouraged to attend.

After filling out the following pages, this checklist document can be attached as a "Supporting Information" document during submission of a manuscript to inform Editors and Reviewers (and eventually readers) that all points of MIRIBEL have been considered.

Supplementary Table 1. Material characterization*

**Question**	**Yes**	**No**
1.1 Are "**best reporting practices**" **available** for the nanomaterial used? For examples, see *Chem. Mater.* **28** (2016) 3535; http://doi.org/10.1021/acs.chemmater.6b01854 and *Chem. Mater.* **29** (2017) 1; http://doi.org/10.1021/acs.chemmater.6b05235	Not applicable	
1.2 If they are available, **are they used**? If not available, ignore this question and proceed to the next one.		
1.3 Are extensive and clear instructions reported detailing all steps of **synthesis** and the resulting **composition** of the nanomaterial? For examples, see *Chem. Mater.* **26** (2014) 1765; http://doi.org/10.1021/cm500632c, and *Chem. Mater.* **26** (2014) 2211; http://doi.org/10.1021/cm5010449. Extensive use of photos, images, and videos are strongly encouraged. For example, see *Chem. Mater.* **28** (2016) 8441; http://doi.org/10.1021/acs.chemmater.6b04639	√	
1.4 Is the **size** (or **dimensions**, if non-spherical) and **shape of** the nanomaterial reported?	√	
1.5 Is the **size dispersity** or **aggregation** of the nanomaterial reported?	√	
1.6 Is the **zeta potential** of the nanomaterial reported?	√	
1.7 Is the **density (mass/volume)** of the nanomaterial reported?	√	
1.8 Is the amount of any **drug loaded** reported? 'Drug' here broadly refers to functional cargos (e.g., proteins, small molecules, nucleic acids).	Not applicable	
1.9 Is the **targeting performance** of the nanomaterial reported, including **amount** of ligand bound to the nanomaterial if the material has been functionalised through addition of targeting ligands?	Not applicable	
1.10 Is the **label signal** per nanomaterial/particle reported? For example, fluorescence signal per particle for fluorescently labelled nanomaterials.	Not applicable	
1.11 If a material property not listed here is varied, has it been **quantified**?	√	
1.12 Were characterizations performed in a **fluid mimicking biological conditions**?	Not applicable	
1.13 Are details of how these parameters were **measured/estimated** provided?	√	
Explanation for **No** (if needed):		

***Ideally, material characterization should be performed in the same biological environment as that in which the study will be conducted. For example, for cell culture studies with nanoparticles, characterization steps would ideally be performed on nanoparticles dispersed in cell culture media. If this is not possible, then characteristics of the dispersant used (e.g., pH, ionic strength) should mimic as much as possible the biological environment being studied.

Supplementary Table 2. Biological characterization*

Question	Yes	No
2.1 Are **cell seeding details**, including **number of cells plated, confluency at start of experiment,** and **time between seeding and experiment** reported?	√	
2.2 If a standardised cell line is used, are the **designation and source** provided?	√	
2.3 Is the **passage number** (total number of times a cell culture has been subcultured) known and reported?	√	
2.4 Is the last instance of **verification of cell line** reported? If no verification has been performed, is the time passed and passage number since acquisition from trusted source (e.g., ATCC or ECACC) reported? For information, see *Science* **347** (2015) 938; http://doi.org/10.1126/science.347.6225.938	√	
2.5 Are the results from **mycoplasma testing** of cell cultures reported?	√	
2.6 Is the **background signal of cells/tissue** reported? (E.g., the fluorescence signal of cells without particles in the case of a flow cytometry experiment.)	√	
2.7 Are **toxicity studies** provided to demonstrate that the material has the expected toxicity, and that the experimental protocol followed does not?	√	
2.8 Are details of media preparation (**type of media, serum,** any **added antibiotics**) provided?	√	
2.9 Is a **justification of the biological model** used provided? For examples for cancer models, see *Cancer Res.* **75** (2015) 4016; http://doi.org/10.1158/0008-5472.CAN-15-1558, and *Mol. Ther.* **20** (2012) 882; http://doi.org/10.1038/mt.2012.73, and *ACS Nano* **11** (2017) 9594; http://doi.org/10.1021/acsnano.7b04855	Not applicable	
2.10 Is characterization of the **biological fluid** (*ex vivo*/*in vitro*) reported? For example, when investigating protein adsorption onto nanoparticles dispersed in blood serum, pertinent aspects of the blood serum should be characterised (e.g., protein concentrations and differences between donors used in study).	Not applicable	
2.11 For **animal experiments**, are the ARRIVE guidelines followed? For details, see *PLOS Biol.* **8** (2010) e1000412; http://doi.org/10.1371/journal.pbio.1000412	Not applicable	
Explanation for **No** (if needed):		

*For *in vitro* experiments (e.g., cell culture), *ex vivo* experiments (e.g., in blood samples), and *in vivo* experiments (e.g., animal models). The questions above that are appropriate depend on the type of experiment conducted.

Supplementary Table 3. Experimental details*

**Question**	**Yes**	**No**
3.1 For cell culture experiments: are **cell culture dimensions** including **type of well**, **volume of added media**, reported? Are cell types (i.e.; adherent vs suspension) and **orientation** (if non-standard) reported?	√	
3.2 Is the **dose of material administered** reported? This is typically provided in nanomaterial mass, volume, number, or surface area added. Is sufficient information reported so that regardless of which one is provided, the other dosage metrics can be calculated (i.e. using the dimensions and density of the nanomaterial)?	√	
3.3 For each type of imaging performed, are details of how **imaging** was performed provided, including details of **shielding, non-uniform image processing**, and any **contrast agents** added?	Not applicable	
3.4 Are details of how the dose was administered provided, including **method of administration, injection location, rate of administration,** and details of **multiple injections**?	Not applicable	
3.5 Is the methodology used to **equalise dosage** provided?	√	
3.6 Is the **delivered dose** to tissues and/or organs (in vivo) reported, as % injected dose per gram of tissue (%ID g^{-1})?	Not applicable	
3.7 Is **mass of each organ/tissue measured** and **mass of material** reported?	Not applicable	
3.8 Are the **signals of cells/tissues with nanomaterials** reported? For instance, for fluorescently labelled nanoparticles, the total number of particles per cell or the fluorescence intensity of particles + cells, at each assessed timepoint.	Not applicable	
3.9 Are **data analysis details**, including **code used** for analysis provided?	√	
3.10 Is the **raw data** or **distribution of values** underlying the reported results provided? For examples, see *R. Soc. Open Sci.* **3** (2016) 150547; http://doi.org/10.1098/rsos.150547, https://opennessinitiative.org/making-your-data-public/, http://journals.plos.org/plosone/s/data-availability, and https://www.nature.com/sdata/policies/repositories	√	
Explanation for **No** (if needed):		

Section S5. References

[1] F. Neese, *WIREs Comput. Mol. Sci.* **2012**, *2*, 73–78.
[2] N. Mardirossian, M. Head-Gordon, *J. Chem. Phys.* **2015**, *142*, 074111.
[3] F. Weigend, R. Ahlrichs, *Phys. Chem. Chem. Phys.* **2005**, *7*, 3297–3305.
[4] F. Weigend, *Phys. Chem. Chem. Phys.* **2006**, *8*, 1057–1065.
[5] V. Barone, M. Cossi, *J. Phys. Chem. A.* **1998**, *102*, 1995–2001.
[6] a) C. Riplinger, F. Neese, *J. Chem. Phys.* **2013**, *138*, 034106; b) C. Riplinger, B. Sandhoefer, A. Hansen, F. Neese, *J. Chem. Phys.* **2013**, *139*, 134101; c) M. Saitow, U. Becker, C. Riplinger, E. F. Valeev, F. Neese, *J. Chem. Phys.* **2017**, *146*, 164105.
[7] a) T. H. Dunning Jr., *J. Chem. Phys.* **1989**, 90, 1007–1023; b) N. B. Balabanov, K. A. Peterson, *J. Chem. Phys.* **2005**, *123*, 064107.
[8] a) F. Weigend, A. Köhn, C. Hättig, *J. Chem. Phys.* **2002**, 116, 3175–3183; b) J. G. Hill, J. A. Platts, *J. Chem. Phys.* **2008**, *129*, 134101.
[9] J. Ho, *Phys. Chem. Chem. Phys.* **2015**, *17*, 2859–2868.
[10] E. F. Pettersen, T. D. Goddard, C. C. Huang, G. S. Couch, D. M. Greenblatt, E. C. Meng, T. E. Ferrin, *J. Comput. Chem.* **2004**, *25*, 1605–1612.
[11] a) G. Galstyan, E.-W. Knapp, *J. Comput. Chem.* **2015**, *36*, 69–78; b) R. Casasnovas, J. Ortega-Castro, J. Donoso, J. Frau, F. Muñoz, *Phys. Chem. Chem. Phys.* **2013**, *15*, 16303–16313.
[12] J. Ho, M. L. Coote, *Theor. Chem. Acc.* **2009**, *125*, 3–21.
[13] H. A. De Abreu, L. Guimarães, H. A. Duarte, *J. Chem. Phys. A.* **2006**, *110*, 7713–7718.
[14] A. Stefánsson, *Environ. Sci. Technol.* **2007**, *41*, 6117–6123.
[15] W. N. Perera, G. Hefter, *Inorg. Chem.* **2003**, *42*, 5917–5923.
[16] V. E. Jackson, A. R. Felmy, D. A. Dixon, *J. Chem. Phys. A.* **2015**, *119*, 2926–2939.
[17] a) F. J. Millero, W. Yao, J. Aicher, *Mar. Chem.* **1995**, *50*, 21–39; b) F. J. Millero, D. J. Hawke, *Mar. Chem.* **1992**, *40*, 19–48.
[18] M. Faria, M. Björnmalm, K. J. Thurecht, S. J. Kent, R. G. Parton, M. Kavallaris, A. P. Johnston, J. J. Gooding, S. R. Corrie, B. J. Boyd, P. Thordarson, A. K. Whittaker, M. M. Stevens, C. A. Prestidge, C. J. H. Porter, W. J. Parak, T. P. Davis, E. J. Crampin, F. Caruso. *Nat. Nanotechnol.* **2018**, *13*, 777–785.

Section S6. Author Contributions

S.P., W.X., and F.C. conceived the ideas, designed, and led the project. All authors performed research and/or analyzed data with intellectual contributions. W.X., S.P., and F.C. drafted the manuscript with intellectual input from all authors. The authors declare no conflict of interest.
